# Nanoenzymes‐Integrated and Microenvironment Self‐Adaptive Hydrogel for the Healing of Burn Injury and Post‐Burn Depression

**DOI:** 10.1002/advs.202413032

**Published:** 2024-12-25

**Authors:** Weitao Zhao, Xi Chen, Ziwei Han, Zengyu Xun, Yilin Qi, Heping Wang, Chang Chen, Zhongying Gong, Xue Xue

**Affiliations:** ^1^ State Key Laboratory of Medicinal Chemical Biology, College of Pharmacy Nankai University Haihe Education Park, 38 Tongyan Road Tianjin 300353 P. R. China; ^2^ Tianjin First Central Hospital, School of Medcine Nankai University No. 24 Fukang Road, Nankai District Tianjin 300192 P. R. China; ^3^ Key Laboratory of Radiopharmacokinetics for Innovative Drugs, Chinese Academy of Medical Sciences, Tianjin Key Laboratory of Radiation Medicine and Molecular Nuclear Medicine, Institute of Radiation Medicine Chinese Academy of Medical Sciences & Peking Union Medical College Tianjin 300192 P. R. China

**Keywords:** burn treatment, depression, hydrogel dressing, inflammation management

## Abstract

Burn injuries often cause prolonged oxidative stress and inflammatory pain due to an initial increase in inflammatory responses, consequently exacerbating depressive disorders and severely impairing patients’ quality of life. The primary function of traditional burn dressings is to prevent infection and facilitate tissue repair. However, these dressings are not intended for the inflammatory pain and depression that often occur during recovery. This study describes a self‐healing hydrogel H@EFCP, which is designed to alleviate inflammatory pain and post‐burn depression in burn injuries. This hydrogel is synthesized through the cross‐linking of carboxymethyl chitosan with borate ester chelates formed from epigallocatechin gallate and 4‐formylphenylboronic acid. The incorporated Prussian blue nanoparticles increase the ability of H@EFCP to regulate the inflammatory process. H@EFCP is effective in the treatment of skin burns by reducing oxidative stress and improving the microenvironment of peripheral inflammation in mice. This modulation consists of a reduction of central nervous system inflammation and the risk of post‐burn depression. Behavioral assays indicate that the hydrogel significantly reduces feelings of despair and anxiety after burns. Consequently, H@EFCP provides a dual‐effect solution for the care and recovery of burn patients, including both burn repair and the associated psychological effects.

## Graphical abstract

This research aimed to mitigate oxidative stress at the burn wound site through the application of a hydrogel dressing, thereby alleviating depression resulting from the activation of central inflammation. The objective is to employ the wound‐peripheral‐central pathway to mitigate pain and depression resulting from inflammation following burn injuries, thereby providing an effective therapeutic outcome for burn patients.

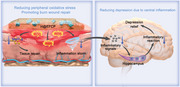

## 1 Introduction

Burns result from the exposure to heat, cold, electricity, chemicals, radiation, or friction. A subsequent complex healing process starts, represented by hemostasis, inflammation, proliferation, and remodeling.^[^ ^1^ ^]^ The intense thermal stimulation from burns causes protein denaturation and tissue damage,^[^ ^2^ ^]^ which in turn leads to the rapid release of inflammatory mediators.^[^ ^3^ ^]^ These mediators not only increase the inflammatory pain^[^ ^4^ ^]^ but also induce peripheral oxidative stress,^[^ ^5^ ^]^ exacerbating central inflammation and potentially leading to post‐burn depression.^[^ ^6^ ^]^ Over half of individuals experiencing chronic pain with long‐standing peripheral inflammation also show depressive symptoms, including fatigue and anhedonia, which may potentially lead to suicidal intents.^[^ ^7^ ^]^ Consequently, it is of the utmost importance to develop an approach that includes an effective repair of burn wounds and allows depressive mood recovery in burn patients.

Hydrogel dressings have recently emerged as a vital approach in the treatment of burns, thanks to their ability to facilitate wound healing and their exceptional biocompatibility.^[^ ^5^ ^]^ Hydrogels are water‐containing polymer networks formed by the cross‐linking of natural or synthetic polymers and they possess the same properties of soft tissues, thus suitable for the production of burn dressings.^[^ ^8^ ^]^ At this stage, the main goals for improving hydrogel dressings for burn treatment are the reduction of wound infection and the promotion of wound healing. For example, Huang et al.^[^ ^9^ ^]^ developed an antimicrobial hydrogel incorporating tobramycin, which is released in response to pH changes in the wound. The significant antimicrobial effect of this hydrogel was confirmed in vitro and in vivo, which is crucial for preventing infections in the burn wound. Xu et al.^[^ ^10^ ^]^ described a magnesium silicate spray that adapts to the inflammatory conditions of the burn wound by releasing magnesium and silicon ions that diminish oxidative stress and accelerate the healing process. Despite these advances, the management of burn‐induced inflammatory pain and central inflammation remains poorly addressed. These aspects are essential for comprehensive burn care, impacting not only the healing of the burn wound, but also the alleviation of post‐burn depression.

Damage‐associated molecular patterns are released by necrotic cells and damaged tissues following burn injury and activate inflammatory pathways, leading to the rapid production of inflammatory mediators by resident immune cells. Then, vasodilation and increased recruitment of additional immune cells into the affected area occur, thus escalating peripheral inflammation.^[^ ^11^ ^]^ Specifically, the inflammatory factors *tnf‐α* and *il‐1β*,^[^ ^12^ ^]^ produced by M1‐type macrophages recruited to the burn wound directly stimulate sensory neurons, resulting in the release of neuropeptides including substance P,^[^ ^13^ ^]^ which activates signaling pathways in the upstream neural network, increasing the perception of pain. Furthermore, the neuroinflammation is intensified by the migration of immune cells and inflammatory mediators across the blood‐brain barrier allowed by the increased peripheral inflammation following a burn injury.^[^ ^14^ ^]^ This enhanced neuroinflammation is involved in the pathogenesis of neuropsychiatric disorders.^[^ ^15^ ^]^ A clear example is inflammatory depression, which is a subtype of depression that is closely associated to the activation of the immune response of both peripheral and central nervous system (CNS). The presence of inflammatory pain signals and the infiltration of peripheral inflammatory signals in the CNS activate microglia, which are the resident immune cells of the brain parenchyma, which in turn release an excess of inflammatory cytokines, including *tnf‐α*, *il‐1β*, and *il‐6*, ultimately resulting in depressive‐like behavior.^[^ ^16^ ^]^

In light of the above, a meticulously crafted hydrogel dressing named H@EFCP is described in this work for the repair of the basement membrane of the burn wound, as well as for the alleviation of inflammatory pain and post‐burn depression that commonly occur after a burn injury. Carboxymethyl chitosan (CMCS) and poly(vinyl alcohol) (PVA) were selected as the main structure of H@EFCP due to their excellent biosafety. The borate ester complex formed by Epigallocatechin gallate (EGCG) and 4‐formylphenylboronic acid (4‐FPBA) was employed as a cross‐linking agent for H@EFCP.^[^ ^17^ ^]^ Concurrently, the hydrogen bonding between PVA and CMCS facilitated the formation of a double cross‐linked network within the hydrogel, thereby enhancing the mechanical properties of H@EFCP. Furthermore, prussian blue nanoparticles (HMPB) were incorporated into H@EFCP with the objective of reducing oxidative stress in the peripheral region and optimizing the inflammatory microenvironment of the wound.^[^ ^18^ ^]^ The boronic ester and Schiff base structure of H@EFCP was degraded in a wound microenvironment with high reactive oxygen species (ROS) and low pH, leading to the release of HMPB and EGCG, which attenuated the inflammatory response of wounds and reduce wound expression of matrix metalloproteinase‐9 (MMP‐9)^[^ ^19^ ^]^ in the wound and promote wound basement membrane repair.^[^ ^20^ ^]^ The objective of this study was to construct dynamically cross‐linked hydrogels with favorable mechanical properties, which would meet the daily activity demands of patients' wound sites on the one hand, and, on the other, to alleviate central inflammation‐induced post‐burn depression by reducing peripheral oxidative stress and inflammatory pain. The results indicate that H@EFCP accelerates the healing of the burn wound and attenuates peripheral oxidative stress and inflammatory pain by reducing inflammation at the wound site. This comprehensive approach further alleviates depression resulting from inflammation in the CNS, paving the way for further investigation into the applicability of H@EFCP.^[^ ^21^ ^]^

## 2 Results and Discussion

### 2.1 Preparation and Characterization of Hydrogels and HMPB

**Scheme 1:**
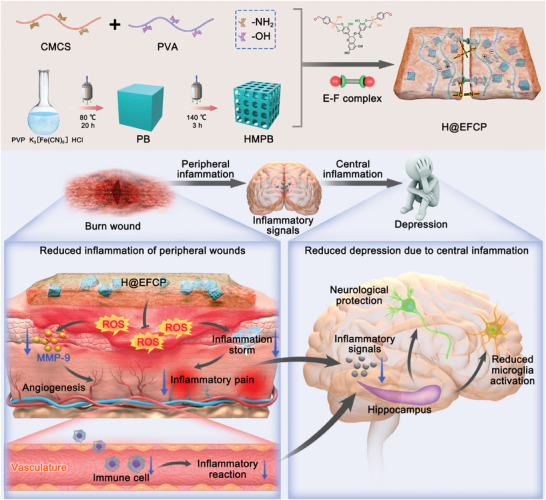
H@EFCP for burn wound repair and post‐burn depression intervention therapy. The hydrogel is formed using CMCS and PVA as the main structure, which is then dynamically crosslinked using an E‐F complex to create H@EFCP. H@EFCP promotes wound healing and attenuates pain response induced by inflammatory microenvironment, thereby reducing the effect of inflammatory infiltration of the peripheral circulation on the brain and inhibiting the depression pathologies and behaviors that occurs in burn patients.

**Figure 1:**
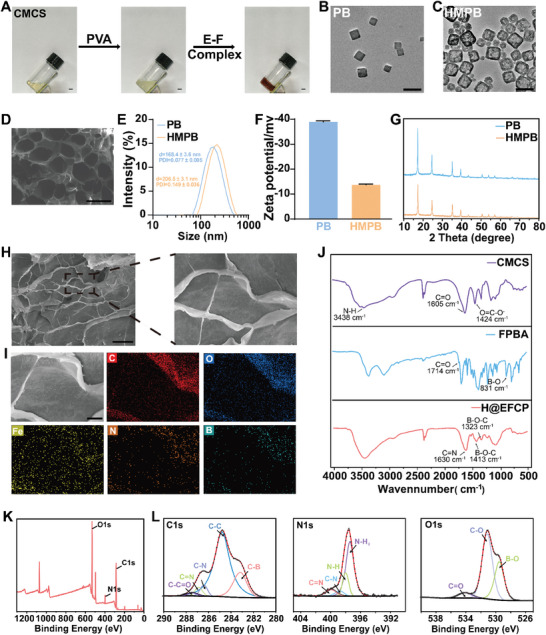
Characterization of H@EFCP and HMPB. A) The optical images of the gelation progress. Upon adding EGCG‐FPBA, CMCS, and PVA mixture experienced a sol‐gel transition. Scale bars, 1 cm. B–C) TEM images of B) PB and C) HMPB nanoparticles. Scale bars, 200 nm. D) SEM images of hydrogels. Scale bar, 100 µm. E) The hydrodynamic sizes of PB and HMPB. Data are expressed as mean ± S.E.M. (*n* = 3). F) Zeta potentials of PB and HMPB. Data are expressed as mean ± S.E.M. (*n* = 3). G) XRD spectra of PB and HMPB nanoparticles. H) Local magnification of the hydrogel SEM image to observe the encapsulation of HMPB. Scale bar, 100 µm. I) The element mapping of H@EFCP hydrogels. Red, blue, yellow, orange, and sky‐blue represented C, O, Fe, N, and B elements. Scale bar, 10 µm. J) FTIR spectra of CMCS, 4‐FPBA, and H@EFCP. Scale bars, 100 µm. K) XPS spectrum of H@EFCP. L) XPS high‐resolution spectrum of C1s, N1s, and O1s.

The synthesis of HMPB and the components of H@EFCP are shown in **Scheme** **1**. The 2:1 ratio of 4‐FPBA to EGCG was selected to prepare the E‐F complex as a cross‐linking agent for the hydrogel.^[^ ^22^ ^]^ The UV‐vis spectra of the EGCG and E‐F complexes showed the presence of new absorption peaks near 325 nm after the formation of the E‐F complexes, indicating the formation of a borate ester bond (Figure S1A, Supporting Information), consistent with the previous report.^[^ ^23^ ^]^ CMCS formed a Schiff base bond with 4‐FPBA as revealed by the UV‐vis spectra and NMR hydrogen spectra of CMCS‐FPBA with the characteristic absorption of the benzene ring on 4‐FPBA (Figure S1B,C, Supporting Information). CMCS was mixed with an aqueous PVA solution for backup (**Figure** **1A**), and then the E‐F complex solution was added for the rapid formation of the hydrogel by dynamic Schiff base binding. The irregular porous network structure of the synthesized hydrogel was observed in the scanning electron microscopy (SEM) images (Figure 1D), potentially providing good air permeability.

The hollow mesoporous HMPB with the properties mentioned above, is involved in the modulation of the oxidative stress microenvironment. Consequently, HMPB was synthesized and integrated into H@EFCP.^[^ ^24^ ^]^ The regular square structure of both PB and HMPB was observed by transmission electron microscopy. The etching process resulted in a distinct cavity structure in the shell of HMPB (Figure 1B,C). Further analysis of the hydrated particle size showed that PB had a hydrodynamic size of 168.4 ± 3.6 nm with a polydispersity coefficient of 0.077 ± 0.005, while HMPB had a hydrodynamic size of 206.5 ± 3.1 nm with a polydispersity index of 0.149 ± 0.036 (Figure 1E). The zeta potentials of both nanoparticles were ‐38.9 mV and ‐13.7 mV, respectively (Figure 1F), which agreed with previous relevant literature^[^ ^25^ ^]^ and confirmed the successful synthesis of HMPB. PB and HMPB nanoparticles possessed identical absorption peaks at 17.3, 24.5, 35.3, 39.4, 43.4, and 57.9 (Figure 1G) as revealed by the powder X‐ray diffractogram pattern, indicating the presence of 200, 220, 400, 420, 422, and 620 crystallographic planes, respectively (JCPDS card 73–0687).^[^ ^26^ ^]^ Concurrently, we investigated the stability of HMPB in aqueous solution and discovered that after 10 days of continuous monitoring (Figure S1E–G, Supporting Information), there was no notable alteration in particle size or potential, suggesting that HMPB can exist stably within the hydrogel. CMCS, PVA, and HMPB were mixed together and set aside to let them cross‐link to form a H@EFCP after the addition of the E‐F complex solution. A uniform dispersion of HMPB on the pores of H@EFCP was observed by SEM, and the presence of C, O, Fe, N, and B elements on the surface of H@EFCP was confirmed by energy dispersive spectroscopy (Figure 1H,I), providing evidence of the successful encapsulation of HMPB. The presence of borate bonds and Schiff bases during the hydrogel formation was confirmed by Fourier transform infrared spectroscopy. Figure 1J showed a broadband absorption peak at 3348 cm^−1^, representing the stretching vibration of N‐H on CMCS.^[^ ^27^ ^]^ The absorption peak at 1714 cm^−1^ is typical of the aldehyde group on FPBA. Furthermore, the absorption peak of H@EFCP at 1630 cm^−1^ confirmed the formation of the Schiff base structure in H@EFCP.^[^ ^28^ ^]^ The presence of absorption peaks at 1413 cm^−1^ and 1323 cm^−1^ was attributed to the asymmetric stretching vibration of the B‐O‐C bond, suggesting the formation of E‐F complexes through borate bonding.^[^ ^29^ ^]^ These findings were confirmed by X‐ray photoelectron spectroscopy (Figure 1K,L), whose peaks at 285.4, 398.4, and 531.6 corresponded to the elements C, N, and O, respectively. Notably, the spectra of C1s and N1s showed the characteristic absorption peaks of C═N at 286.8 and 399.9,^[^ ^30^ ^]^ as well as absorption peaks of B‐O bonds in the O1s spectra, providing further evidence of the presence of borate ester bonds and Schiff base structures in H@EFCP.^[^ ^31^ ^]^ In conclusion, the results presented above demonstrated the successful synthesis of H@EFCP through the formation of dynamic chemical bonds.

### 2.2 Mechanical Properties and Responsive Degradability of H@EFCP

**Figure 2:**
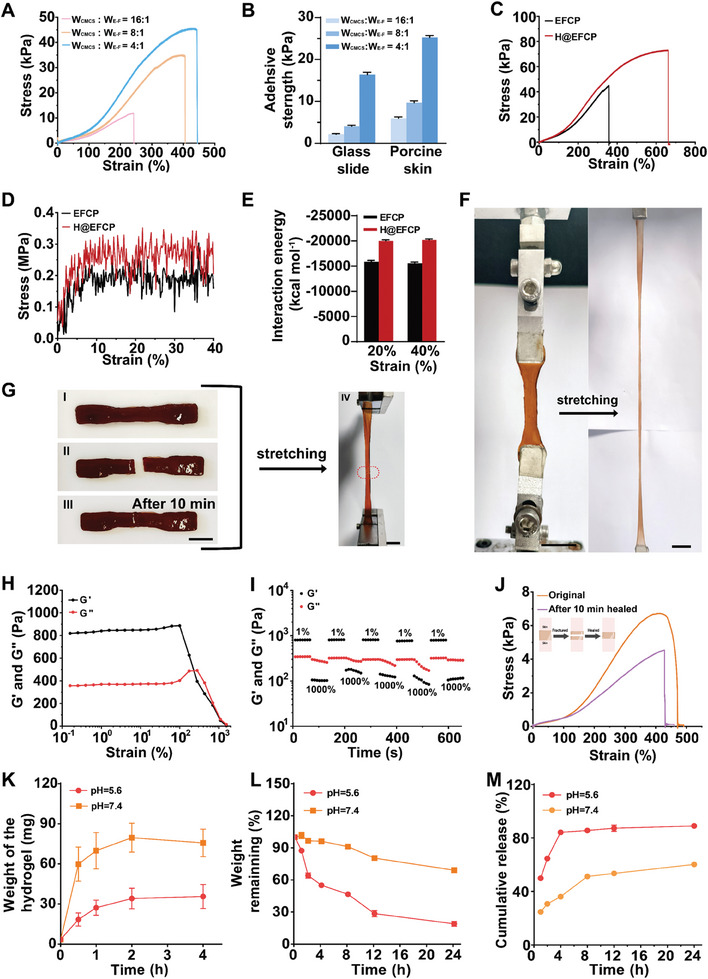
H@EFCP has excellent mechanical properties and responds to complex and irregular burn wound. A) Tensile stress‐strain curves of EFCP containing different ratios of E‐F complexes. B) The adhesion strength of H@EFCP containing different ratios of E‐F complexes to porcine skin tissue and glass slide. Data are expressed as mean ± S.E.M. (*n* = 3). C) Tensile stress‐strain curves of EFCP and H@EFCP (W_CMCS_: W_E‐F_ = 4:1). D) Stress‐strain curves for H@EFCP and EFCP molecular dynamics simulations. E) Interaction energy statistics of H@EFCP and EFCP under different strain conditions. Data are expressed as mean ± S.E.M. (*n* = 3). F) Macroscopic stretching images of hydrogels. Scale bars, 1 cm (W_CMCS_: W_E‐F_ = 4:1). G) Photographs of the macroscopic healing capacities of H@EFCP. The original state (i), damaged state (ii), hydrogel healed for 10 min (iii), and stretching after hydrogel healed (iv). Scale bars, 1 cm (W_CMCS_: W_E‐F_ = 4:1). H) Strain sweep of the hydrogel with the strain ranging from 0.01% to 1500% (1 Hz, W_CMCS_: W_E‐F_ = 4:1). I) G′ and G′′ of H@EFCP during five cycles between 1% and 1000% strain (W_CMCS_: W_E‐F_ = 4:1). J) Adhesive strength of incisional porcine skin bonded by H@EFCP before damage and after healing for 10 min (W_CMCS_: W_E‐F_ = 4:1). K) Swelling behavior of H@EFCP in pH 7.4 and 5.6 at 37 °C Data are expressed as mean ± S.E.M. (*n* = 3, W_CMCS_: W_E‐F_ = 4:1). L) Degradation behavior of H@EFCP in vitro with pH 7.4 and 5.6 at 37 °C Data are expressed as mean ± S.E.M. (*n* = 3, W_CMCS_: W_E‐F_ = 4:1). M) Releasing curves of EGCG from H@EFCP with pH 7.4 and 5.6 at 37 °C. Data are expressed as mean ± S.E.M. (*n* = 3, W_CMCS_: W_E‐F_ = 4:1).

The optimal mechanical properties of hydrogel dressings are of paramount importance to effectively address emergency situations and facilitate the repair of burn wounds. In the case of burn patients, the favorable adhesion and stretchability of burn dressings afford protection for the wound from external stimuli, whilst simultaneously reducing the risk of secondary injury due to the effects of daily activities. Therefore, mechanical property tests, including tensile strength, adhesion, and self‐healing assessments, were initially performed on HMPB‐free hydrogel (EFCP). The abundance of hydroxyl groups on PVA enables the formation of hydrogen bonds with the amino groups on CMCS, thereby enhancing the tensile strength of EFCP. As illustrated in Figure S1D (Supporting Information) and **Figure** **2A**, the tensile strength of EFCP in the absence of PVA is less than 10 kPa, with an elongation of less than 250%. However, following the incorporation of PVA, a notable enhancement in the tensile strength of EFCP was observed, accompanied by a gradual increase in tensile stress and elongation at break with the addition of E‐F complex. Moreover, the adhesion strength of EFCP with different E‐F complexes (W_CMCS_:W_E‐F complex_ = 16:1, 8:1, and 4:1) were 5.98 ± 0.57 kPa, 16.40 ± 0.92 kPa, and 25.26 ± 0.79 kPa, respectively to skin tissues, and 2.18 ± 0.25 kPa, 4.08 ± 0.39 kPa, and 9.08 ± 0.79 kPa, respectively to glass (Figure 2B). This phenomenon is primarily ascribed to the prevalence of amino and hydroxyl groups on CMCS and PVA, which arise from the formation of multiple bonding interactions with skin tissue proteins. Furthermore, the impact of water absorption on the adhesion effect of H@EFCP was investigated to simulate the alteration in hydrogel adhesion following the absorption of wound exudate. As illustrated in Figure S1H (Supporting Information), the adhesion effect of the hydrogel sample remained largely unchanged after 15 minutes of water absorption. The macroscopic images demonstrated that H@EFCP was capable of bearing a certain degree of weight after adhering to pigskin. This indicates that the adhesion effect of H@EFCP does not undergo significant alteration after absorbing a specific volume of exudate. The tensile tests conducted on EFCP and H@EFCP revealed that H@EFCP demonstrated superior tensile stress and elongation at break (Figure 2C) compared to the EFCP. Consequently, molecular dynamics simulations were conducted to examine the alteration in the interaction energy between HMPB, CMCS and PVA. The simulation box for tensile testing was constructed using the molecular structures that comprise H@EFCP (Figure S2, Supporting Information) and subjected to geometry optimization and dynamic equilibrium treatments (Figure S3A,B, Supporting Information). The results demonstrate that H@EFCP exhibits a heightened stress response during stretching (Figure 2D). Furthermore, the internal interaction energies of the H@EFCP and EFCP models during stretching were quantified. H@EFCP exhibited a higher interaction energy compared to EFCP (Figure 2E), indicating the potential for HMPB to facilitate enhanced interactions within the model, thereby improving the mechanical responsiveness of the hydrogel samples. The H@EFCP model continued to stretch until the strain reached 1000%, and H@EFCP still exhibited a higher stress response (Figure S3C, Supporting Information). This finding is also consistent with the results of our practical tests (Figure 2C). The macroscopic stretching image of the H@EFCP in the actual test is presented in Figure 2F. These results suggest that the addition of HMPB induces an enhancement of the hydrogel's internal interactions and improves the tensile strength of the hydrogel. Subsequently, compression tests were performed on H@EFCP samples. A comparison of the hydrogel morphology before and after compression revealed that H@EFCP efficiently returned to its original morphology after the release of the pressure, indicating the good compression resistance (Figure S1I, Supporting Information). These results indicated that H@EFCP possessed exceptional mechanical properties and optimal tissue adhesion, thereby facilitating complete wound coverage. Hydrogels based on borate bond cross‐linking and dynamic bonds such as Schiff bases typically possess exceptional self‐healing ability. Next, a macroscopic healing test and rheological study were performed to evaluate the self‐healing ability of H@EFCP. H@EFCP was cut in half, and the two halves were placed together and left at room temperature. After 10 min, H@EFCP was stretched, showing that it was healed and without fissures (Figure 2G). Subsequently, strain amplitude scanning was performed on H@EFCP. Figure 2H shows that the energy storage modulus (G′) and loss modulus (G′′) intersect at strains lower than 1000%, which was the critical point for hydrogel collapse. Hence, a small strain of 1% and a large strain of 1000% were selected for cyclic testing. At a small strain G′ surpassed G′′ during the small strain, while G′ became lower than G′′ when the strain was switched to 1000%, indicating the destruction of the hydrogel network. G′ again became larger than G′′ when returning to a strain of 1%, indicating the recovery of the hydrogel network. No significant change in the magnitude of G′ and G′′ was observed after five cycles, demonstrating that the H@EFCP structure, based on dynamic bond crosslinking, remained stable and exhibited excellent mechanical self‐healing properties (Figure 2I). The self‐healing properties of H@EFCP were evaluated using two fresh pig skin pieces to assess the ability to bond reopened tissue wounds. The two skin pieces were joined using H@EFCP, and then the H@EFCP was cut at the joining location. Subsequently, the two parts were reconnected, and after 10 minutes, the reconnected pig skin pieces were stretched. The results revealed that the average tensile strength of the reconnected pieces was restored to 64% of the initial tensile strength (Figure 2J). These findings indicated the remarkable self‐healing abilities of H@EFCP as well as the ability to adapt to the deformation of the wound, thereby preventing further damage following joint movement.

The removal of exudate around the wound is a crucial step in the promotion of wound healing. Therefore, the swelling efficiency of the hydrogel at different pH conditions was analyzed (Figure 2K). The weight of H@EFCP continued to increase due to water absorption, and the average weight of the H@EFCP after 4 h was ≈35 mg and 77 mg at pH 5.6 and 7.4 after 4 h, respectively. The ability of H@EFCP to collapse in acidic environments was due to the Schiff base and borate bonding, leading to a lower hydrogel weight at pH 5.6. A similar trend was observed in the in vitro degradation studies (Figure 2L), where H@EFCP showed a higher degradation rate in an acidic medium. Finally, the release of EGCG due to the decomposition of H@EFCP was assessed, indicating that the release was faster at a relatively lower pH compared to the release in a solution with a pH of 7.4 (Figure 2M). These experimental results demonstrated that H@EFCP possessed excellent hygroscopicity and effectively removed wound exudate to protect the burn wound. Furthermore, H@EFCP performed a controlled release of EGCG at the site of the wound. To conclude, H@EFCP possessed excellent mechanical properties, adhesion, self‐healing ability, and degradation behavior, adapting to diverse burn wound deformations and effectively protecting the burn wound.

### 2.3 Antimicrobial and Antioxidant Properties of H@EFCP

**Figure 3:**
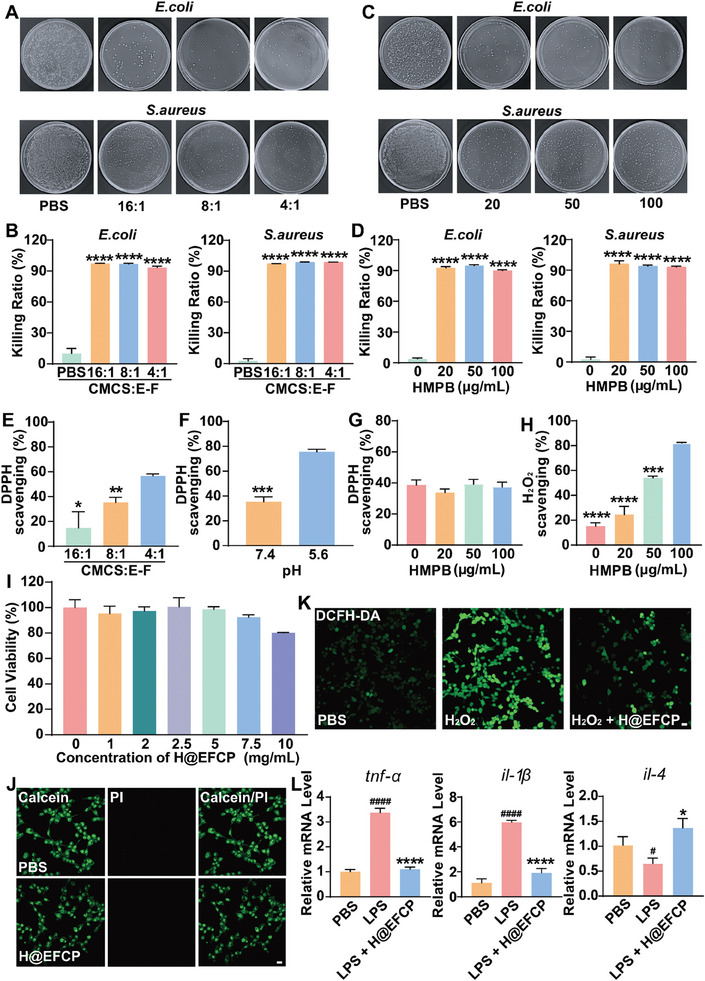
H@EFCP exhibits superior properties on antimicrobial and antioxidant activities in vitro. A) Images of surviving bacterial (*E. coli* and *S. aureus*) clones on agarose plates after contact with PBS and hydrogel with different E‐F complex ratios without HMPB (W_CMCS_:W_E‐F complex_ = 16:1, 8:1, and 4:1). B) Corresponding bacteria‐killing efficiency against *E. coli* and *S. aureus*. in (A). Data are expressed as mean ± S.E.M (*n* = 3, one‐way ANOVA and Sidak's multiple comparison tests, ****p < 0.0001 versus the PBS group). C) Images of bacteria exposed to PBS and H@EFCP containing different concentrations of HMPB surviving on culture plates (0 µg mL^−1^, 20 µg mL^−1^, 50 µg mL^−1^, and 100 µg mL^−1^). D) Corresponding bacteria‐killing efficiency against *E. coli* and *S. aureus*. in (C). Data are expressed as mean ± S.E.M (*n* = 3, one‐way ANOVA and Sidak's multiple comparison tests, ****p < 0.0001 versus the 0 µg mL^−1^ of HMPB group). E) DPPH scavenging capabilities of H@EFCP containing different ratios of E‐F Complexes. Data are expressed as mean ± S.E.M (*n* = 3, one‐way ANOVA and Sidak's multiple comparison tests, *p < 0.05, **p < 0.01 versus the W_CMCS_:W_E‐F complex_ = 4:1 group). F) DPPH scavenging capabilities of H@EFCP at different pH. Data are expressed as mean ± S.E.M (*n* = 3, two‐tailed unpaired Student's *t*‐test, ***p < 0.001 versus the pH = 5.6 group). G) DPPH scavenging capabilities of hydrogels containing different HMPB concentrations. Data are expressed as mean ± S.E.M (*n* = 3). H) H_2_O_2_ decomposition by H@EFCP with different HMPB concentrations. Data are expressed as mean ± S.E.M (*n* = 3, one‐way ANOVA and Sidak's multiple comparison tests, ***p < 0.001, ****p < 0.0001 versus the 100 µg/mL of HMPB group). I) Cell viability of NIH3T3 cells after incubation in the leaching solution with different concentrations for 48 h. Data are expressed as mean ± S.E.M (*n* = 3). J) Live/Dead staining of NIH3T3 cells after incubation in a leaching solution of the H@EFCP (5 mg mL^−1^) for 48 h. Scale bar, 20 µm. K) The alleviation of oxidative stress in NIH3T3 was monitored via a DCFH‐DA after different treatments. Scale bar, 20 µm. L) Expression of relevant inflammatory cytokines, *tnf‐α*, *il‐1β* and *il‐4*. Data are expressed as mean ± S.E.M (*n* = 3, one‐way ANOVA and Sidak's multiple comparison tests, *p < 0.05, ****p < 0.0001 versus the LPS‐treated group, ^#^ *p* < 0.05, ^####^ *p* < 0.0001 versus the PBS group,).

The burn wound is frequently susceptible to bacterial infection, leading to an exacerbated inflammatory response that inhibits the healing process.^[^ ^32^ ^]^ Consequently, a dressing possessing antimicrobial properties prevents bacterial contamination and facilitates wound healing. Previous studies showed that CMCS has inherent antimicrobial effects.^[^ ^33^ ^]^ This study evaluated the antibacterial properties of H@EFCP, which contained different concentrations of E‐F complexes, by surface antibacterial activity testing against *Escherichia coli* (*E. coli*), which is a Gram‐negative bacterium, and *Staphylococcus aureus* (*S. aureus*), which is a Gram‐positive bacterium. The bacteria and hydrogel samples were co‐incubated at 37 °C for 6 h, and then, the same quantity of bacterial culture from each experimental group was placed into agarose plates and incubated for 12 h. The groups treated with hydrogels containing different concentrations of E‐F complex showed a decrease in the number of bacterial clones on the agar plates compared with the control group treated with PBS (**Figure** **3**A; Figure S4A,B, Supporting Information). No change in the inhibition rate of bacterial growth was observed for each group of hydrogels containing different concentrations of E‐F complex, whose rates were 97.3%, 96.8%, and 93.5% for *E. coli* and 97.3%, 98.9%, and 98.9% for *S. aureus*, respectively (Figure 3B). Similarly, H@EFCP with different concentrations of HMPB was tested and the results showed that each hydrogel group containing various concentrations of HMPB had significantly less bacterial clones than the control group (PBS) (Figure 3C; Figure S4C,D, Supporting Information), but no significant difference was found among the hydrogel groups, whose inhibition rates were 92.7%, 94.9%, and 90.1% for *E. coli* and 96.1%, 94.0%, and 93.1% for *S. aureus*, respectively (Figure 3D). The above results demonstrated that the antimicrobial properties of H@EFCP were mainly provided by CMCS.

The immune system is activated in response to the acute injury due to the burn wound, generating a large amount of ROS,^[^ ^34^ ^]^ which further trigger oxidative stress in the wound microenvironment, exacerbating inflammatory pain and peripheral inflammation.^[^ ^35^ ^]^ Therefore, ROS should be effectively scavenged to reduce oxidative stress and facilitate wound healing. The antioxidant properties of H@EFCP were investigated by in vitro DPPH free radical scavenging assay (Figure 3E), revealing that the scavenging effect on H@EFCP increased from an initial 14.7% to 56.6% with an increase in the content of E‐F complex in the hydrogel. Next, H@EFCP samples with a CMCS: E‐F complex ratio of 8:1 were constructed since H@EFCP decomposed in acidic environments and the scavenging ability was assessed at different pH environments. H@EFCP exhibited a higher scavenging activity under acidic conditions, with a scavenging rate of 75.5% (Figure 3F), which suggested that the release of EGCG was gradually increased with the decomposition of the hydrogel under acidic conditions, resulting in an increased scavenging of DPPH radicals. Moreover, the ability of HMPB in the hydrogel to promote the decomposition of DPPH was assessed using H@EFCP samples containing different concentrations of HMPB, and the results revealed no significant change in scavenging DPPH (Figure 3G). However, the degree of scavenging H_2_O_2_ increased with the increased HMPB concentration when H_2_O_2_ was used as the oxygen radical medium. H_2_O_2_ degradation was the highest (82.0%) when HMPB concentration reached 100 µg mL^−1^ compared to other HMPB concentrations (Figure 3H). Additionally, the comparison of scavenging H_2_O_2_ by H@EFCP (HMPB 100 µg mL^−1^) and EFCP separately in the H_2_O_2_ medium revealed that the degree of scavenging H_2_O_2_ by H@EFCP was significantly higher than that by EFCP (Figure S5A, Supporting Information), consistent with the previously described results. As illustrated in Figure S5B (Supporting Information), HMPB has the capacity to inhibit the reduction of NBT, thereby indicating that it possesses SOD enzyme activity. With regard to H@EFCP, as the pH decreases, H@EFCP displays a higher inhibition rate (Figure S5C, Supporting Information). The aforementioned results demonstrate that H@EFCP has excellent antioxidant capacity and is capable of scavenging exogenous ROS. Therefore, H@EFCP effectively neutralized the complex oxidative stress at the wound site, suggesting the ability of H@EFCP in improving the inflammatory microenvironment of the burn wound.

### 2.4 Effects of H@EFCP on Inflammatory Inhibition In Vitro

Biocompatibility of the dressing is necessary to perform an effective wound repair. An excellent biocompatibility contributes to the prevention of secondary damages to the wounds.^[^ ^36^ ^]^ The cytotoxicity and biocompatibility of H@EFCP were assessed by the MTT assay and live/dead staining. H@EFCP was co‐incubated with a cell culture solution (without cells) for 24 h, and NIH3T3 cells were cultured in the collected leachate for 48 h. The survival rate of the cells in the experimental groups was higher than 95% except for the group treated with 10 mg mL^−1^ and 7.5 mg mL^−1^ leachate (Figure 3I). Therefore, the concentration of 5 mg mL^−1^ was chosen for subsequent experiments. No significant difference in alive and dead cells between the H@EFCP group and the experimental group, as revealed by the live/dead staining (Figure 3J). The MTT and live/dead staining experiments at 24 h gave similar results, such as no difference was found in the number of live/dead cells between the control and H@EFCP groups (Figure S5D,E, Supporting Information).

The removal of ROS from the burn wound site directly reduces inflammation in the periphery. The ability of H@EFCP to scavenge cellular ROS was assessed by incubating the hydrogel leachate with H_2_O_2_‐treated NIH3T3 cells and ROS scavenging activity was assessed using DCFH‐DA. The intracellular ROS levels were significantly reduced in the H@EFCP group compared to the H_2_O_2_‐treated positive control group, whose fluorescence intensity was comparable to that of the PBS group (Figure 3K; Figure S5F, Supporting Information). Similarly, an analogous methodology was utilized to treat human neuroblastoma cells (SH‐SY5Y). The findings demonstrated that the fluorescence intensity of cells treated with H@EFCP was markedly diminished in comparison to cells treated with H2O2 (Figure S5G,H, Supporting Information). This indicates that H@EFCP displays a notable capacity to eliminate ROS in an in vitro setting. The promotion of M2 macrophage activation in the burn wound helps the suppression of the inflammatory microenvironment.^[^ ^37^ ^]^ Thus, RAW264.7 macrophage polarization to the M1 state was obtained using lipopolysaccharide (LPS), then incubated with H@EFCP, and the expression of inflammatory factors was measured. H@EFCP significantly reduced the expression of the pro‐inflammatory factors *tnf‐α* and *il‐1β* ^[^ ^38^ ^]^ but increased the expression of the anti‐inflammatory factor *il‐4* ^[^ ^39^ ^]^ compared to the control group (Figure 3L). This effect was primarily attributed to the release of EGCG and HMPB from the H@EFCP, which attenuated oxidative stress by reducing ROS levels.

### 2.5 Wound Repair Ability of H@EFCP in a Mouse Burn Model

**Figure 4:**
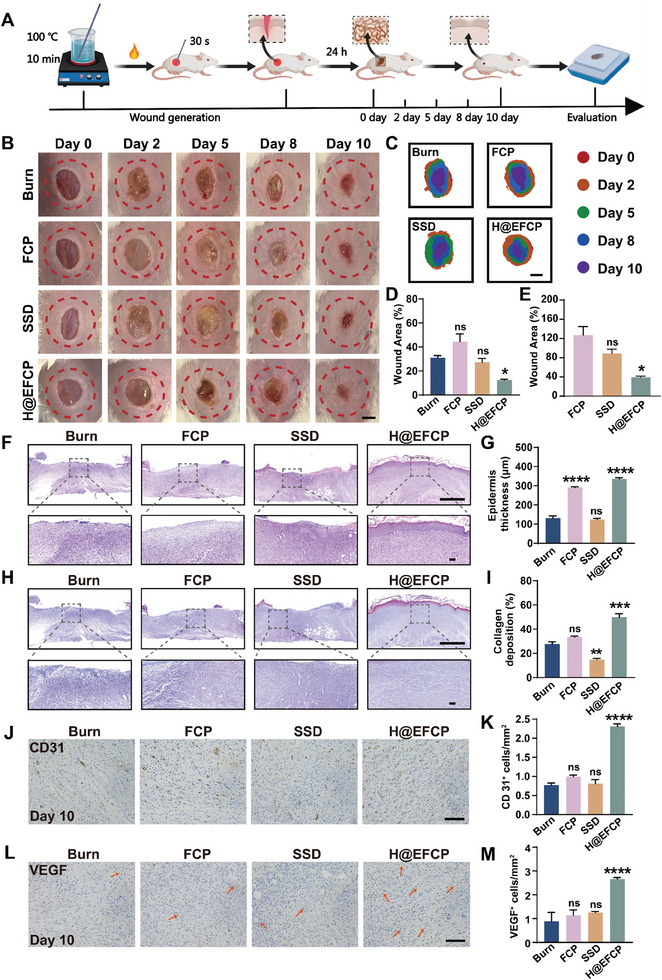
Efficacy of H@EFCP in burn wound repair. A) The schematic establishment and treatment of the burn wound. B) Representative photographs of the wounds treated with different samples. Scale bar, 3 mm. C) Wound traces of the healing process for 10 days. Scale bar, 4 mm. D) Relative area of the wound after 10 days of treatment. Data are expressed as mean ± S.E.M. (*n* = 4, one‐way ANOVA and Sidak's multiple comparison tests, ns, no significance, *p < 0.05 versus the burn group). E) The area of wounds in the treatment group is relative to the control group after 10 days of treatment. Data are expressed as mean ± S.E.M. (*n* = 4, one‐way ANOVA and Sidak's multiple comparison tests, ns, no significance, *p < 0.05 versus the FCP group). F) H&E staining of wound sections in all groups on day 10. Scale bars, 1 mm (original images); 100 µm (zoomed‐in images). G) Quantification of Epidermis thickness on day 10. Data are expressed as mean ± S.E.M. (*n* = 4, one‐way ANOVA and Sidak's multiple comparison tests, ns, no significance, ****p < 0.0001 versus the burn group). H) MT images of wounds on day 10. Scale bars, 1 mm (original images); 100 µm (zoomed‐in images). I) Collagen accumulation on day 10 based on MT staining. Data are expressed as mean ± S.E.M. (*n* = 4, one‐way ANOVA and Sidak's multiple comparison tests, ns, no significance, **p < 0.01, ***p < 0.001 versus the burn group). J‐M) IHC staining and quantification of (J), (K) CD31, and (L), (M) VEGF. Scale bars,100 µm. Data are expressed as mean ± S.E.M. (*n* = 4, one‐way ANOVA and Sidak's multiple comparison tests, ns, no significance, ****p < 0.0001 versus the burn group).

Encouraged by the good in vitro biological properties of H@EFCP, the healing effect of H@EFCP on a mouse burn wound model was evaluated. The mouse burn model was obtained by heating a glass rod to 100 °C and placing it on the skin of BALB/c mice for 30 s (**Figure** **4A**).^[^ ^40^ ^]^ After forming a wound with a diameter of 10 mm, the wound was debrided and coated with FCP (without EGCG and HMPB), silver sulfadiazine disulfiram (SSD),^[^ ^41^ ^]^ or H@EFCP at specific intervals (Figure 4B; Figure S6, Supporting Information). In addition, wound healing traces were drawn based on representative photographs of the wounds of each group at different time points to point out the macroscopic therapeutic effect (Figure 4C). At the beginning of the treatment, the wounds of the mice treated without H@EFCP increased in size due to the movement of the mice, while those of the mice treated with H@EFCP showed a clear tendency to heal at day 5 and were accompanied by scab formation. The wounds treated with H@EFCP were almost completely healed after 10 days of treatment, whereas the wounds remained unclosed in the mice treated with other methods. As obtained by measuring the area of the wound at different experimental times based on the wound photographs and assessing the average area of the wound in each group after 10 days of treatment. The average area of the burn group and the FCP group were 31.2% and 44.4%, respectively. The average area of the wound in the group treated with SSD was 27.3%, while it was only 12.4% in the group treated with H@EFCP (Figure 4D). Moreover, the change in the wound area of each group relative to the burn group was measured on day 10 and the results showed that the H@EFCP group had an average wound reduction of 60.7% compared to that in the burn group, which was better than the reduction measured in the FCP and SSD groups, as shown in Figure 4E. These results indicated that the H@EFCP group healed faster and had excellent burn wound healing.

Wound healing is a complex process associated with hemostasis, anti‐inflammation, cell proliferation, and extracellular matrix remodeling.^[^ ^42^ ^]^ Thus, the healing effect was evaluated using H&E and MT staining. The H&E staining results showed that the H@EFCP group had less inflammatory cells and increased epidermal layer thickness at the wound site compared to the other treatment groups (Figure 4F,G). This result suggested that H@EFCP promoted the formation of the epidermal layer at the wound site, which was beneficial to wound healing. In addition, collagen deposition at the wound site detected by MT staining revealed that the H@EFCP group had greater collagen deposition, which reached 49.8% (Figure 4H,I), suggesting that H@EFCP promoted collagen deposition in the epithelial tissue, further promoting the reconstruction of the basement membrane of the burn wound.

Angiogenesis is essential for wound healing since blood vessels provide nutrients to healing‐related cells and sustain the growth of the neoplastic granulation tissue. The angiogenic effects of H@EFCP were assessed by immunohistochemical staining for platelet endothelial cell adhesion molecule‐1 (CD31) and vascular endothelial growth factor (VEGF). The experimental group treated with H@EFCP showed higher CD31 and VEGF protein expression than the other groups (Figure 4J–M). Thus, these results suggested that H@EFCP promoted the growth of traumatic granulation tissue, collagen deposition, and blood vessel formation, thus allowing the healing of the burn wound.

### 2.6 Modulation of the Traumatic Inflammatory Environment and Relieve of the Inflammatory Pain by H@EFCP

**Figure 5:**
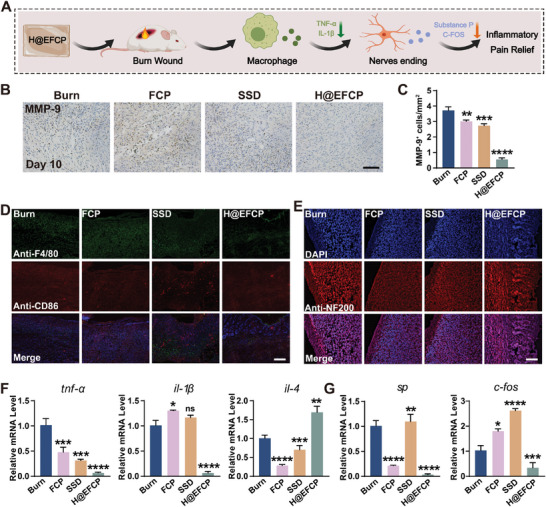
H@EFCP remodels of the inflammatory microenvironment and modulation of inflammatory pain. A) Schematic representation of pain caused by the inflammatory microenvironment of the wound. B‐C) IHC staining and quantification of MMP‐9. Scale bar,100 µm. Data are expressed as mean ± S.E.M. (*n* = 4, one‐way ANOVA and Sidak's multiple comparison tests, **p < 0.01, ***p < 0.001, ****p < 0.0001 versus the burn group). D) Immunofluorescence staining of F4/80/CD86 in wound tissue. Scale bar, 200 µm. E) Immunofluorescence staining of NF200 on day 10. Scale bar,200 µm. F) *Tnf‐α*, *il‐1β*, and *il‐4* mRNA levels in the wound tissue on day 10. Data are expressed as mean ± S.E.M. (*n* = 4, one‐way ANOVA and Sidak's multiple comparison tests, ns, no significance, *p < 0.05, **p < 0.01, ***p < 0.001, ****p < 0.0001 versus the burn group). G) The *sp* and *c‐fos* mRNA levels on day 10. Data are expressed as mean ± S.E.M. (*n* = 4, one‐way ANOVA and Sidak's multiple comparison tests, *p < 0.05, **p < 0.01, ***p < 0.001, ****p < 0.0001 versus the burn group).

The activated immune system increases the peripheral inflammation following burn‐related tissue damage and stimulates pain receptors to trigger an inflammatory pain response.^[^ ^43^ ^]^ Inflammatory factors (e.g., *tnf‐α* and *il‐1β*) produced by activated macrophages during the inflammatory response act on the injury receptors, leading to an increase in their sensitivity, causing an increase in the expression of c‐fos in traumatized primary sensory neurons.^[^ ^44^ ^]^ Furthermore, substance P (sp) released from primary afferent fibers is also increased,^[^ ^13^ ^]^ which further enhances pain signals transmitted to secondary neurons in the spinal cord and brainstem, resulting in the perception of pain after tissue injury (**Figure** **5A**). Therefore, the pain response at the site of injury can be reduced by controlling the inflammatory microenvironment in the burn wound. This was performed by measuring the expression of MMP‐9 in the burn wound since it is closely related to inflammation, which was significantly lower in H@EFCP group than in the other groups, with a relative positive cell count of 0.56% (Figure 5B,C). H@EFCP reduces MMP‐9 expression by releasing EGCG,^[^ ^45^ ^]^ which promotes collagen deposition and accelerates wound repair, thus suggesting the ability of H@EFCP to improve the inflammatory microenvironment of the injured tissue. The regulatory effect of H@EFCP on the inflammatory microenvironment at the injured site was further investigated by performing immunofluorescence staining for F4/80 and CD86 in the peri‐wound tissues after different treatments (Figure 5D). The results revealed that H@EFCP reduced the number of M1‐type macrophages at the inflammatory microenvironment of the wound. Pain in the wound tissue is reduced by repairing nerve endings.^[^ ^46^ ^]^ Therefore, the nerves in the peri‐wound tissue were labelled after treatment to observe the nerve repair at the injured site by the detection of the NF200 protein.^[^ ^47^ ^]^ NF200 protein expression was increased in the H@EFCP group compared with that in the other three groups, indicating that this hydrogel was able to activate the neurons in the wound tissue to start the repair state, inducing neuron growth and alleviating the pain sensation (Figure 5E). Moreover, an increased CD90 expression was found in H@EFCP group, suggesting an increase in the number of mesenchymal stem cells (MSC) in the wound tissue treated with H@EFCP (Figure S7A, Supporting Information). Thus, the effect of H@EFCP on nerve repair might be due to the impact of MSCs. Next, the expression of relevant inflammatory and pain factors in the wound was analyzed after 10 days of treatment by RT‐qPCR. H@EFCP significantly decreased *tnf‐α* and *il‐1β* expression and increased *il‐4* expression (Figure 5F,G). The expression of substance P and c‐Fos was considerably reduced in H@EFCP group compared with the burn group, which was respectively 1/9 and 1/3 times that of the burn group. The expression of inflammatory factors in the normal and burn groups was also examined, revealing that the burn group exhibited a remarkable increased expression of inflammatory factors (Figure S7B, Supporting Information). The above results suggested that H@EFCP reduced inflammatory pain perception and promoted traumatic nerve repair by modulating the inflammatory microenvironment in the traumatic tissue and reducing the expression of pain factors. Furthermore, the safety evaluation of H@EFCP demonstrated that no damage or histopathological lesions were observed in the tissue sections of the heart, liver, spleen, lungs, and kidneys (Figure S7C, Supporting Information). Concomitantly, there was no notable alteration in body weight between the H@EFCP cohort and the Sham group (Figure S7D, Supporting Information). An in vitro hemolysis test demonstrated that H@EFCP does not induce hemolysis (Figure S7E, Supporting Information). These findings suggest that H@EFCP exerts no significant toxic effects and can be further utilized as a burn dressing.

### 2.7 Reduction of Post‐Burn Depression in Mice by H@EFCP

Clinical reports revealed that burn victims, especially those with severe burns, often experience depression, disinterest, and loss of confidence,^[^ ^48^ ^]^ with depression usually associated with high levels of peripheral inflammation.^[^ ^49^ ^]^ Burn‐induced traumatic injury promotes an immune response that increases immune cells and cytokines (including *il‐1β*, *tnf‐α*, and *il‐6*) in the peripheral blood and injured tissues, which enter the brain, increase brain inflammation, and affect cognitive function.^[^ ^16^ ^]^ Peripheral inflammation alters the number, morphology, and function of microglia, consequently causing depression.^[^ ^50^ ^]^ HLA‐DR expression (MHC class II proteins, which are surface markers of activated microglia) is higher in the hippocampus of depressed patients than in controls.^[^ ^51^ ^]^ Injured CNS is often accompanied by an increased number of microglia, as well as altered morphology (changes in thickness and length) and expression of the activation marker^[^ ^52^ ^]^ ionized calcium‐binding articulation molecule 1 (IBA1). Our results showed that white blood cell count was reduced, and the proportion of neutrophils increased in the post‐burn mouse model. In addition, a reduction in the levels of alkaline phosphatase (ALP) and blood urea nitrogen (BUN) was found in the blood, indicating the presence of an inflammatory infiltrate in the periphery of the post‐burn mice. However, the number of neutrophils was reduced after the treatment with H@EFCP and ALP and BUN returned to normal levels, suggesting that H@EFCP effectively alleviated peripheral inflammation (Figure S8A–D, Supporting Information).

**Figure 6:**
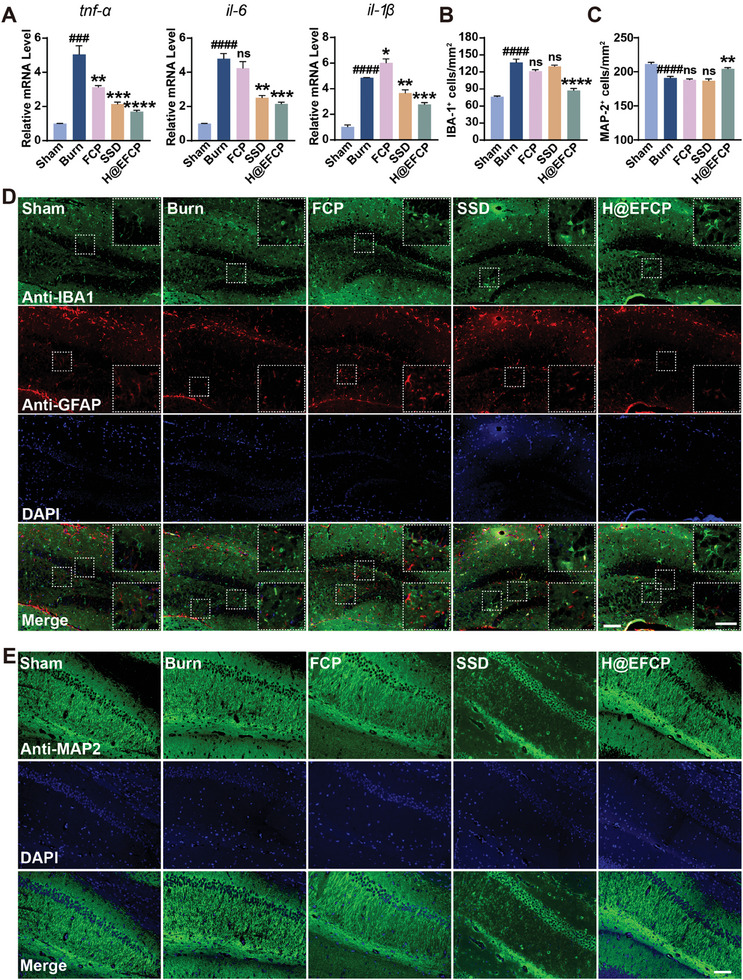
H@EFCP reduces central inflammation levels through modulation of peripheral inflammation. A) Tnf‐α, il‐1β, and il‐6 mRNA levels in brain tissue. Data are expressed as mean ± S.E.M. (*n* = 5, one‐way ANOVA and Sidak's multiple comparison tests, ns, no significance, *p < 0.05, **p < 0.01, ***p < 0.001, ****p < 0.0001 versus the burn group, ^###^ *p* < 0.001, ^####^ *p* < 0.0001 versus the sham group). B) The number of IBA‐1 positive microglia in the DG region. Data are expressed as mean ± S.E.M. (*n* = 3, one‐way ANOVA and Sidak's multiple comparison tests, ns, no significance, ****p < 0.0001 versus the burn group, ^####^ *p* < 0.0001 versus the sham group). C) The number of MAP‐2 positive neurons in the CA1 region. Data are expressed as mean ± S.E.M. (*n* = 3, one‐way ANOVA and Sidak's multiple comparison tests, ns, no significance, **p < 0.01 versus the burn group, ^####^ *p* < 0.0001 versus the sham group). D) Immunofluorescence staining of IBA‐1 and GFAP in the DG region of the hippocampus. Scale bars, 100 µm (original images); 50 µm (zoomed‐in images). E) Immunofluorescence staining of MAP‐2 in the CA1 region of the hippocampus. Scale bar, 100 µm.

**Figure 7:**
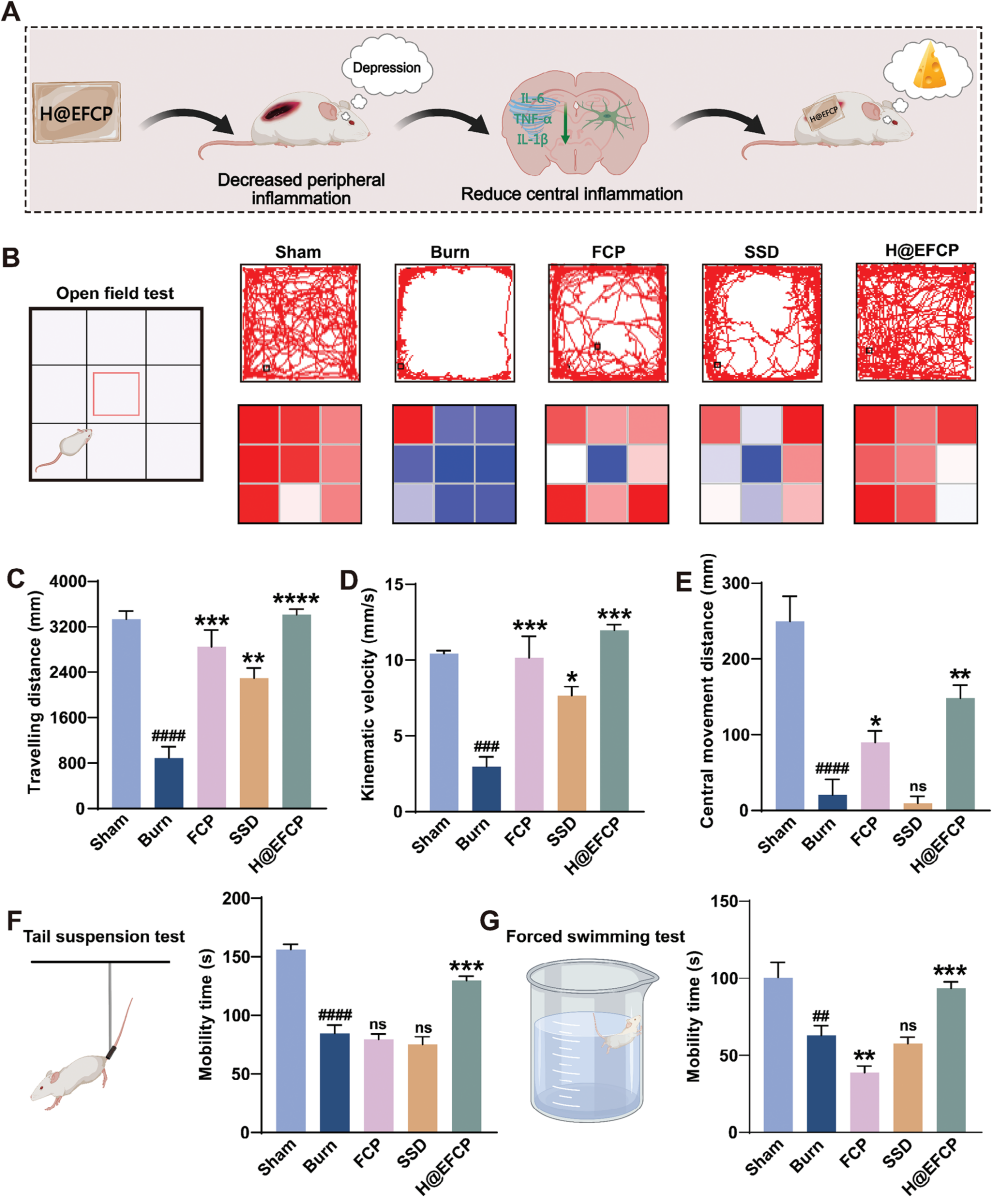
H@EFCP inhibits the depressive behaviors after burn injury. A) Peripheral inflammation caused by the burn wound leads to elevated expression of inflammatory factors in the brain promoting depression. B) Movement tracking images and heatmaps of mice in the OFT. C) Total distance of movement in the OFT. Data are expressed as mean ± S.E.M. (*n* = 5, one‐way ANOVA and Sidak's multiple comparison tests, **p < 0.01, ***p < 0.001, ****p < 0.0001 versus the burn group, ^####^ *p* < 0.0001 versus the sham group). D) Average speed of movement in the OFT. Data are expressed as mean ± S.E.M. (*n* = 5, one‐way ANOVA and Sidak's multiple comparison tests, *p < 0.05, ***p < 0.001 versus the burn group, ^###^ *p* < 0.001 versus the sham group). E) Total distance traveled in the OFT center area. Data are expressed as mean ± S.E.M. (*n* = 5, one‐way ANOVA and Sidak's multiple comparison tests, ns, no significance, *p < 0.05, **p < 0.01 versus the burn group, ^####^ *p* < 0.0001 versus the sham group). F) Mobility time in the tail suspension test. Data are expressed as mean ± S.E.M. (*n* = 5, one‐way ANOVA and Sidak's multiple comparison tests, ns, no significance, ***p < 0.001 versus the burn group, ^####^ *p* < 0.0001 versus the sham group). G) Mobility time in the forced swim test. Data are expressed as mean ± S.E.M. (*n* = 5, one‐way ANOVA and Sidak's multiple comparison tests, ns, no significance, **p < 0.01, ***p < 0.001 versus the burn group, ^##^ *p* < 0.01 versus the sham group).

Activation of peripheral inflammation typically results in an intensified central inflammatory response in the brain, which is associated with the onset of depression. Therefore, the regulation of central inflammation is of paramount importance for an effective treatment of post‐burn depression. The expression of inflammatory factors in the brain was assessed by RT‐qPCR to evaluate the effect of different treatments on central brain inflammation in the mouse burn model. The results showed that H@EFCP markedly reduced the expression of *tnf‐α*, *il‐6*, and *il‐1β* pro‐inflammatory mediators, in comparison to the burn group (**Figure** **6A**), thus reducing the inflammation in the brain, which may potentially exert a beneficial effect on the alleviation of depressive symptoms. Next, the reactive markers IBA1 and glial fibrillary acidic protein (GFAP) of microglia and astrocytes in the hippocampus were evaluated since the development of depression is highly correlated with the expression of inflammatory factors in the hippocampus.^[^ ^53^ ^]^ The results showed that microglia in the burn group had significantly larger cell bodies and shorter branches than those in the sham group; in contrast, astrocytes in the burn group had considerably enlarged cell bodies and more branches (Figure 6D). However, the treatment with H@EFCP resulted in morphological changes resembling those of the sham group in both microglia and astrocytes, such as microglia showed increased branching, while astrocytes showed a reduction in cell body size. Moreover, the average expression of IBA1 in H@EFCP group was significantly lower compared to that of the sham group (Figure 6B), indicating that H@EFCP inhibited the activation of microglia and astrocytes. The morphology of neurons in the hippocampus was also assessed using the marker microtubule associated protein‐2 (MAP‐2), and the results revealed that neuronal axons were shorter in the burn group than in the sham group. However, H@EFCP restored the axon length of neurons, and the number of neurons was increased (Figure 6C,E). The number of Nissl bodies in the mice after burns was also decreased, which was restored after treatment with H@EFCP (Figure S8E, Supporting Information). This suggests that H@EFCP can reverse depressive‐like behaviors in animals by exerting neuroprotective effects. Both human and mouse model studies have consistently demonstrated a strong correlation between depression and the decreased expression of brain‐derived neurotrophic factor (BDNF). Immunohistochemical staining for BDNF also showed that treatment with H@EFCP restored BDNF levels in the brains of burned mice and reduced the production of burn‐induced depression (Figure S8F, Supporting Information). In conclusion, treatment with H@EFCP reduced inflammatory activation on microglia and astrocytes by attenuating peripheral inflammatory levels and reducing the expression of central inflammatory factors. These findings have positive implications for the alleviation of depression caused by central inflammation (**Figure** **7A**).

Next, depression in the burn mouse model after different treatments was assessed by behavioral tests considering the reduction of the central inflammation exerted by H@EFCP on the brain. The open field test (OFT) revealed that the trajectories for the burn, FCP, and SSD groups were predominantly localized in the limbic region, in contrast to the sham group (Figure 7B). Similarly, total mouse movement distance and average mouse speed were significantly reduced in the burn, FCP and SSD groups, suggesting the potential presence of depression (Figure 7C,D). The analysis and counting of the hot zones of the mice's behavior in the field trials revealed an increase in the distance of movement in the central region in the mice treated with H@EFCP compared to the mice in the other treatment groups, indicating a reduction of anxiety and depression (Figure 7E). The tail suspension test and forced swimming test revealed a significant reduction in the active time of mice in the burn, FCP and SSD groups compared to that in the sham group, suggesting that the inflammatory response induced by burn injury might lead to the onset of core psychiatric symptoms such as anxiety and depression. However, the mice showed increased activity times after the treatment with H@EFCP, demonstrating that the hydrogel effectively alleviated the anxiety and depression‐like behavior associated with post‐burn injury (Figure 7F,G). These findings suggested that increased central inflammation, driven by peripheral inflammation due to burn injury contributed to mood depression in mice. Psychotropic drugs have been traditionally used to alleviate post‐burn depression, often leading to patient dependence. However, our research showed the effectiveness of H@EFCP on reducing central inflammation induced by peripheral oxidative stress, consequently reducing anxiety and depressive behavior in mice after burn injury, providing a new therapeutic approach for the treatment of post‐burn depression.

## 3 Conclusion

The healing of the burn wound is a complex and prolonged process.^[^ ^54^ ^]^ Thermal irritation and damage significantly alter the wound microenvironment, leading to the development of inflammation,^[^ ^55^ ^]^ which compromises the integrity of the basement membrane and exacerbates symptoms such as pain and itching.^[^ ^56^ ^]^ Furthermore, the inflammatory response after a burn injury contributes to the increase of pain and psychological distress.^[^ ^48^ ^]^ Consequently, promoting burn wound healing and simultaneously attenuating patients' negative emotional responses represents a promising avenue for improving burn care. The repair of a burn wound is composed of four different stages: hemostasis, inflammation, proliferation, and remodeling.^[^ ^54a^ ^]^ The presence of inflammation in the wound inhibits the healing process and leads to the interaction of inflammatory factors with nerve endings, resulting in the transmission of pain signals.^[^ ^34^ ^]^ Furthermore, the simultaneous activation of the peripheral immune system increases inflammation in the brain, thereby exacerbating central inflammation that contributes to depressive symptoms.^[^ ^57^ ^]^ Therefore, the modulation of peripheral oxidative stress might be a promising approach to regulate inflammation during wound repair, thereby attenuating CNS response to inflammation and reducing the risk of inflammatory pain and depression in patients.

The objective of this study was to develop a hydrogel dressing designed to reduce the risk of depression in burn patients through the wound repair process. H@EFCP was based on chelates formed by EGCG and FPBA, cross‐linked with CMCS to form a hydrogel, and PVA was added to ameliorate the mechanical properties. This hydrogel has an excellent antimicrobial effect, proper tissue adhesion, and self‐healing ability to adapt to complex wounds and prevent local stress injury due to the dynamic chemical bonding within the hydrogel itself. Moreover, HMPB loaded in the hydrogel, as well as EGCG released after degradation, regulated the inflammatory microenvironment in the wound, reduced the expression of inflammatory factors, attenuated the release of MMP‐9, regulated the repair of wound vascularity and wound basement membrane, reduced the expression of relevant pain factors, and promoted the regeneration of wound nerve endings. H@EFCP alleviated the exacerbation of central inflammation induced by peripheral oxidative stress, thereby reducing the incidence of depression associated with central inflammation. This approach avoids the risks associated with the use of neurological medications following burn injuries. In conclusion, this study presented a new and pertinent strategy using H@EFCP that facilitates burn wound repair and reduces the risk of inflammatory pain and depression in patients recovering from burns.

The relationship between inflammatory pain and depression caused by peripheral damage is primarily related to oxidative stress at the wound site and the presence of continuous exogenous stimuli in daily life. Therefore, in the future, we hope to construct a hydrogel dressing that has both good mechanical properties and the ability to reduce oxidative stress at the wound site. On the one hand, the mechanical properties of hydrogel will be relied upon to reduce the psychological burden on patients caused by the constant stimulation of the wound in their daily lives. On the other hand, by reducing the inflammatory response in the wound, the risk of inflammatory depression in patients is reduced. This approach has a positive impact on the management of complex peripheral wounds (e.g., burns, diabetic foot ulcers, etc.) which cause psychological distress to patients.

## 4 Experimental Section

### Fabrication of the Hydrogel

First, 6% CMCS solution and 8% PVA solution were configured, and HMPB was dissolved in PVA solution to make the final concentration of 20 µg mL^−1^, 50 µg mL^−1^, and 100 µg mL^−1^; after that, the same volume of PVA and CMCS were taken and stirred well at room temperature. Finally, E‐F complex solution was added to the above mixture and stirred well, and then it was left to stand for 3 min at room temperature to obtain H@EFCP hydrogel.

### Mechanical Characterization of the Hydrogel

The mechanical characterization of the hydrogels was conducted using an Instron mechanical tester, equipped with a calibrated 5‐N load cell. All samples were cut into rectangular pieces, measuring 25 mm × 10 mm × 2 mm, and the tensile test was set at a tensile rate of 30 mm min^−1^. At least three specimens were tested for each sample.

### In Vitro Assessment of Inflammation

The capacity of the hydrogel to scavenge ROS at the cellular level was evaluated through the utilization of a DCFH‐DA probe. In brief, NIH3T3 cells were seeded onto a 24‐well plate and incubated for 12 h. Subsequently, the cells were treated with a hydrogel leachate containing 1 µmol mL^−1^ H₂O₂, with cells treated with only 1 µmol mL^−1^ H₂O₂ serving as a positive control and cells treated with PBS serving as a negative control. Following a six‐hour treatment period, the cells were rinsed with PBS and stained with DCFH‐DA for 20 min. The same procedure was carried out on SH‐SY5Y cells. Finally, the cells were imaged using CLSM.

RAW 264.7 cells (1×10^5^ cells mL^−1^) were cultured in 6‐well plates for 12 h. Next, LPS (1 µg mL^−1^) was incubated with cells for another 24 h. The cells were subsequently treated with or without H@EFCP hydrogel extract with 5 mg mL^−1^. After 24 h stimulation, total RNA was extracted using Trizol reagent, then RNA was transferred to the aqueous phase through chloroform, precipitated by isopropanol, and finally separated by centrifugation. The obtained RNA was quantified by NanoDrop 8000. A certain amount of RNA was used to invert into cDNA and further quantified by quantitative real‐time polymerase chain reaction (RT‐qPCR). β‐actin functioned as endogenous housekeeping gene to normalize the corresponding mRNA. The mRNA expression level was calculated based on comparative Ct method (2^−ΔΔCt^). All of the primers were designed by Primer‐BLAST (National Center for Biotechnology Information) and listed in Table S1 (Supporting Information).

### Animals Experiments

All animals were purchased from Beijing Vital River Laboratory Animal Technology Co. Ltd. All the animal experiments were conducted according to the Regulations for the Administration of Affairs Concerning Experimental Animals (Tianjin, revised in June 2004) and the Animal Ethics Committee of Nankai University (approval number: 2021‐SYDWLL‐000457).

### In Vivo Skin Burns Wound Healing Experiments

A burn model was established to evaluate the healing ability of hydrogel. Briefly, 8‐week‐old female BALB/c mice (SPF grade) were anesthetized, and the dorsal hair was removed. Metal hoops will be customized in hot water at 100 °C for 10 min. The preheated metal iron bar was pressed on the dorsal hairless skin for 10 seconds to create a skin burn wound. After establishing the burn models for 24 h, the dead tissue was removed using skin biopsy punches with scissors. Then, mice were randomly divided into three groups: control group (without any treatment), FCP hydrogel group (without E‐F complex and HMPB), SSD group (burn ointment, silver sulfadiazine), and H@EFCP hydrogel group, and change treatment medications every two days. The healing state of the wounds was recorded by digital photographs on days 0, 2, 5, 8, and 10. The wound contraction of each group was accurately measured using professional software (ImageJ), and the wound closure was calculated as follows.

(1) $$\mathrm{Wound}\;\mathrm{area}\;\left(\%\right)=\frac{S_{0}-S_{t}}{S_{0}}\times 100\%$$

where S_0_ and S_t_ were the wound areas on day t and day 0, respectively.

### Statistical Analysis

Results were analyzed by using GraphPad Prism 7 software. Differences between the two groups were assessed using unpaired *t*‐tests. For multiple comparisons, statistical significance was analyzed using one‐way analysis of variance (ANOVA), followed by Sidak's post‐hoc test, which was used when comparing all the conditions. The level of statistical significance was set at *p* < 0.05. *p < 0.05, ^#^ *p* < 0.05 were considered significant, and **p < 0.01, ***p < 0.001, ****p < 0.0001, ^##^ *p* < 0.01, ^###^ *p* < 0.001, ^####^ *p* < 0.0001 were considered highly significant. Unless otherwise indicated, all data were expressed as mean ± standard error of the mean (S.E.M).

## Conflict of Interest

The authors declare no conflict of interest.

## Supporting information



Supporting Information

## Data Availability

The data that support the findings of this study are available on request from the corresponding author. The data are not publicly available due to privacy or ethical restrictions.
