# Supplementary material for: Nanoenzymes‐Integrated and Microenvironment Self‐Adaptive Hydrogel for the Healing of Burn Injury and Post‐Burn Depression
Source: Adv Sci (Weinh). 2024 Dec 25;12(7):2413032. doi: 10.1002/advs.202413032 (PMC11831452; doi:10.1002/advs.202413032)
Supplement: Supplementary file 1 — Supporting Information [file ADVS-12-2413032-s001.docx]

Supporting Information

**Nanoenzymes-integrated and microenvironment self-adaptive hydrogel for the healing of burn injury and post-burn depression**

*Weitao Zhao, Xi Chen, Ziwei Han, Zengyu Xun, Yilin Qi, Heping Wang, Chang Chen,* Zhongying Gong*, *Xue Xue**

W. Zhao, X. Chen, Z. Han, Z. Xun, Y. Qi, Dr. H. Wang C. Chen and Prof. X. Xue

State Key Laboratory of Medicinal Chemical Biology, College of Pharmacy, Nankai University, Haihe Education Park, 38 Tongyan Road, Tianjin 300353, P. R. China

E-mail: [xuexue@nankai.edu.cn](mailto:xuexue@nankai.edu.cn)

Z. Gong
Current address: Tianjin First Central Hospital,School of Medcine, Nankai University, No. 24 Fukang Road, Nankai District, Tianjin 300192, P. R. China.

E-mail: [13212121250@163.com](file:///C:\Users\Zhao%20Weitao\Desktop\文章\第十六版\13212121250@163.com)

H. Wang

Current address: Key Laboratory of Radiopharmacokinetics for Innovative Drugs, Chinese Academy of Medical Sciences, Tianjin Key Laboratory of Radiation Medicine and Molecular Nuclear Medicine, Institute of Radiation Medicine, Chinese Academy of Medical Sciences & Peking Union Medical College, Tianjin 300192, P. R. China.

**MATERIALS AND METHODS**

**Reagents and materials**

Carboxymethyl chitosan (CMCS, Macklin), PVA (Mw: 89000-98000 kDa, Macklin), Epigallocatechin gallate (EGCG, Aladdin), 4-Acetylphenylboronic acid (FPBA, Macklin), Ferricyanide (K_3_[Fe(CN)_6_] ·3H_2_O, Aladdin), Poly(vinylpyrrolidone) (PVP K30, Macklin), H_2_O_2_ solution (30 wt%, Macklin). Hydrogen chloride solution (HCl, 37%, Macklin), 2,7-dichlorofuor-escin diacetate (DCFH-DA, Beijing Dingguo Changsheng Biotechnology Co., Ltd.), High glucose Dulbecco’s modified Eagle’s medium (DMEM, Gibco), 1% penicillin-streptomycin (PS, Gibco), 0.25% trypsin–EDTA, phosphate and phosphate-buffered saline (PBS), Fetal bovine serum (FBS, Gibco).

**Instrumentation**

SEM images were obtained on a JSM-7800F SEM (JEOL Company Ltd., Japan) operated at an accelerating voltage of 30 kV. TEM images and element mapping were obtained on a JEM 2800 TEM (JEOL Company Ltd., Japan) operated at an accelerating voltage of 200 kV. FTIR spectra were recorded on a Bruker TENSOR 37 FTIR analyzer (Bruker, Germany). UV-vis spectra were determined by a Shimadzu 2550 UV-vis scanning spectrophotometer (Shimadzu, Japan). Nuclear magnetic resonance hydrogen spectrum was determined by a digital NMR spectrometer (AVANCE NEO 400MHz). Rheological properties were determined using a TA rheometer (DHR-2). All the tensile tests were performed using an Instron mechanical tester equipped with a calibrated 5-N load cell (model 3365). The absorbance was detected using the microplate reader (Tecan, Austria). The fluorescent images were observed by a V5800 inverted fluorescence microscope (Viyee, China) and LSM 800 with an Airyscan confocal laser scanning fluorescence microscope (Zeiss, Germany). The particle size was determined by dynamic light scattering (Malvern, United Kingdom).

**Preparation of HMPB nanoparticles**

HMPB was synthesized according to a previous report with a few modifications. First, 135 mg of K_3_[Fe(CN)_6_] and 9 g of PVP K30 were added to 120 mL of HCl solution (0.01 M) and stirred together for 30 minutes to obtain a clear solution. The solution was transferred to the autoclave and heated at 80 °C for 20 hours without stirring. After that, the PBs were obtained by centrifugation (12000 rpm, 10 min) and washed with ethanol and deionized (DI) water three times.

Then, PB (40 mg) and PVP (200 mg) were dissolved in 40 mL of HCl solution (1 M) under magnetic stirring for 3 hours. HMPB was obtained by heating the solution in an autoclave at 140 °C for 3 h. The HMPB were collected by centrifugation (12000 rpm, 10 min), washed with ethanol, and deionized (DI) water thrice.

**Preparation of EGCG-FPBA complexation (E-F complex)**

EGCG (24 mg) was dissolved in 4 mL H_2_O at a 6 mg/mL. FPBA (16 mg) was added to the solution at a 4 mg/mL concentration. The mixture was mixed by a vortex mixer for 3 minutes and rested for 15 minutes to drive complex formation. The ultimate molar ratio of FPBA and EGCG was 2.

**Characterizations**

The morphology of the PB and HMPB were characterized by transmission electron microscopy (TEM). The hydrogels were freeze-dried and cut into pieces for characterization by the scanning electron microscope (SEM). Fourier transform infrared spectroscopy (FTIR) was used to detect the forces forming the hydrogels. The swelling ratios of the hydrogels were represented through the weight changes before and after their water immersion (swelling ratio = weight after immersion in water/weight before immersion in water×100%).

**Adhesive properties of the hydrogel**

Fresh pork slices were procured from the market and subsequently sliced into 2 × 3 cm pieces. Similarly, glass slides of identical dimensions were prepared and subjected to lap-shear tests to assess the adhesion strength of the H@EFCP hydrogel on diverse surfaces. The hydrogel was distributed across the surface of the pork skin and glass slide, ensuring that the gel adhesion area was 1 cm × 1 cm. Ultimately, lap-shear tests were conducted to quantify the adhesion strength.

**Rheological properties of the hydrogel**

The rheological properties of hydrogels were tested using a TA rheometer (DHR-2). Hydrogel precursor solution was placed between the parallel plates of 20 mm diameter and with a gap of 1000 μm. The hydrogel samples' storage modulus (G') and loss modulus (G'') were examined after the hydrogel precursor solution was first placed between parallel plates with a diameter of 20 mm and a gap of 1000 micrometers. At the constant frequency of 1 HZ and 1% of strain, the time sweep tests were performed at 37 ℃.

**Self-Healing behavior of the hydrogel**

Initially, a rheometer was employed to assess the self-healing behavior by transitioning the amplitude oscillation strain from a minor strain (1.0%, 60 seconds) to a substantial strain (1000%, 60 seconds) at a constant angular frequency (1 rads^-1^) over five cycles.

Besides, the hydrogel sheet was divided into two sections, which were then re-glued and left to stand for a period of 10 minutes. Finally, the self-healing properties were tested by subjecting the material to macroscopic stretching.

At last, fresh pork skin, cut in half, was affixed with hydrogel, left for 30 minutes, and then the hydrogel joint was severed, allowing the two parts to be repositioned together. Subsequently, the bond strength of the re-glued skin was assessed after an additional 10 minutes.

**Molecular dynamics simulation methods**

In this work, all the molecular simulations (MD) are performed using the Large-scale Atomic/Molecular Massively Parallel Simulator (LAMMPS-2Aug2023). The model structures in the MD simulation are presented by OVITO. The CVFF force field is utilized to account for the interactions in CMCS, PVA, E-F complex, and HMPB. Among them, 100 CMCS, 100 PVA, 200 E-F complex and 8 PB nanoparticles are selected for the construction of hybrid model. Periodic boundary conditions were applied in all three directions and the steep descent method was used to minimize the energy in each system. The Velocity Verlet algorithm was adopted for integration. Lorentz- Berthelot mixing rules were adopted for van der Waals interactions for different kinds of atoms. The cut-off distances for electrostatic interactions and van der Waals interactions were 1 nm.

The models for tensile simulation are achieved by melt quenching. After these models are constructed, the whole system is equilibrated in the constant temperature and pressure ensemble (NPT) at 400 K and 1 atm for 0.5 ns, during which the uniform density of the models is achieved. Subsequently, the system is quenched by bringing the temperature and pressure to 298 K and 1 atm for 0.5 ns and equilibrated for 0.5 ns. During the tensile simulation, the whole system is first equilibrated in the NPT at 298 K and 1 atm for 1.0 ns. During this relaxation, the stress inside the models can be released. Afterwards, the pressure is controlled to be 0 atm in the z directions, and the engineering strain rate is 1 × e^-6^ ~1× e^-4^ Å^-1^ in the x direction for 40 ps.

***In Vitro* Swelling Test**

For the *in vitro* swelling test, hydrogel blocks of the same weight (5 mg) after lyophilization were placed in 5 mL of citric acid-Na_2_HPO_4_ buffer solution (pH 5.6 and 7.8, respectively). When the preset time interval was reached, the hydrogels were removed from the vials and the hydrogels were weighed.

***In vitro* degradation behavior**

For the *in vitro* degradation test, Hydrogel blocks of the same weight (500 mg) were tested in 5 mL of citric acid-Na_2_HPO_4_ buffer solutions (pH = 5.6 and 7.8, respectively) at constant temperature (37 °C) with shaking at 100 rpm, respectively. At the predetermined time point, the hydrogel sample was taken out, excess water was removed, and the sample was weighed. The following equation then defined the weight remaining (%) of hydrogels:

Weight remaining of the hydrogel (%) = W_t_ / W_0_×100%, W_0,_ and W_t_ are the weight of the initial hydrogel and the weight of the remaining hydrogels after degradation at different time points, respectively.

**Antibacterial activity**

The antibacterial activity of the hydrogel was tested using Escherichia coli (*E. coli*) and Staphylococcus aureus (*S. aureus*) as test organisms. In brief, the sterilized hydrogels were added to a 48-well plate, and then 200 µL of bacterial suspensions (in PBS, 1×10 CFU/mL) were added to the surface of the hydrogel. Subsequently, the 48-well plate was incubated at 37 °C for 4 h in a relatively humidified atmosphere. Subsequently, 300 µL of sterilized PBS was added to each well in order to re-suspend any bacterial survivors. A 200 μL aliquot of the bacterial suspension (1 × 10^6^ CFU/mL) was added to a 300 μL solution of PBS, mixed thoroughly, and used as a control. Following 24 hour incubation period at 37 °C, the colony-forming units (CFU) on the Petri dish were enumerated, and the growth of bacteria on each agar plate was documented. Tests were repeated three times for each group. The killing ratios of bacteria were calculated using the following equation:

$$\text{Killing ratio\%=}\frac{\text{cell count of control group-survival count on the hydrogels}}{\text{cell count of control group}}\text{×100\%}$$

**Antioxidant efficiency of the hydrogel**

Hydrogels' antioxidant efficiency was evaluated by scavenging the stable DPPH free radical and reactive oxygen species assay. First, the hydrogels were cut into homogeneity using a tissue grinder. 100 µM DPPH and different amounts of the hydrogel samples were dispersed in 3.0 mL ethanol. The mixture was stirred and incubated in a dark place at 37 °C for half an hour. Then, the absorbance of each sample was calculated using a UV-Vis spectrophotometer. The following formula calculated the degradation of DPPH:

$$\text{DPPH scavenging \%=}\frac{\text{A}_{\text{Blank}}\text{-}\text{A}_{\text{Hydrogel}}}{\text{A}_{\text{Blank}}}\text{×100\%}$$

Where A_Blank_ and A_Hydrogel_ are the absorption of the blank (DPPH + ethanol) and the absorption of the hydrogel (DPPH + ethanol + hydrogel), respectively.

**The ROS-scavenging ability of** **the hydrogel**

Titanyl sulfate (0.03 M) was used for evaluating the ROS-scavenging ability of the hydrogel. H_2_O_2_ solution (1 mM, 3 mL) was incubated with different contents of hydrogels in buffers of different pH for 30 minutes. The supernatants (100 μL) were collected, and 30 μL titanyl sulfate was added. Subsequently, the H_2_O_2_ scavenging rate was determined by measuring the absorbance spectra of the above mixture of solutions.

**SOD-like activity of the hydrogel**

The NBT (nitroblue tetrazolium) photoreduction method was employed to ascertain the SOD enzyme activity of H@EFCP. The solution containing NBT was initially combined with the PBS and HMPB samples, and the absorbance of the samples at 550 nm was subsequently measured following a 30 minutes coincubation at 37 °C. This was done to ascertain the intrinsic SOD-like activity of HMPB. Subsequently, the SOD-like activity of H@EFCP under disparate pH conditions was ascertained by combining the NBT solution with H@EFCP in Tris-HCl buffer solutions of varying pH (5.6 and 7.8, respectively) and measuring the absorbance of the samples at 550 nm.

**Cytocompatibility evaluation of the hydrogel**

The cytotoxicity of H@EFCP hydrogel was evaluated by MTT assay. NIH3T3 cells were seeded in a 96-well plate at 2500 cells/well density. After NIH3T3 cells were cultured for 24 h, different concentrations (0-10 mg/mL) of H@EFCP hydrogel extract were incubated with cells for another 24 h. Then, 100 μL MTT solution was replaced with the medium and further incubated for 4 h. Eventually, the medium was replaced by 150 μL DMSO per well, and the plate was shaken for 0.5 h. A microplate reader was used to read the absorbance value at 570 nm of each well.

Live/Dead staining was conducted with NIH3T3 cells. NIH3T3 cells (1×10^4^ cells/mL) were cultured in 24-well plates for 24 h. Then, the medium was replaced with H@EFCP hydrogel extract and further incubated for 24 h. Eventually, staining with 500 μL of calcein-AM/propidium iodide dye for 15 minutes, cells were observed under a fluorescent microscope for the green (492 nm) and red (545 nm) fluorescence.

***In Vitro* Hemocompatibility Test**

To obtain erythrocytes for the hemolytic activity assay, mouse blood was subjected to centrifugation at 116 g for a period of 10 minutes. The erythrocytes were then washed three times with PBS, after which the purified erythrocytes were further diluted to a final concentration of 5% (v/v). Subsequently, The hydrogel (50 mg) was then mixed with the erythrocyte stock solution (1 mL) and shaken at 100 rpm for 1 h in an incubator at 37 °C. A 0.1% Triton X-100 solution was employed as a positive control, while a PBS buffer solution was used as a negative control. Following centrifugation at 1,000 rpm for 10 minutes, the supernatant (100 µL) was transferred to a 96-well microplate. The absorbance of the solution was then measured at 540 nm. The percentage hemolysis was calculated using the following formula:

$$\text{Hemolysis}\text{ \%=}\frac{\text{A}_{\text{Hydrogel}}-\text{A}_{\text{PBS}}}{\text{A}_{\text{Triton X-100}}-\text{A}_{\text{PBS}}}\text{×100\%}$$

where A_Hydrogel_ is the absorbance value of supernatant treated with determined sample. A_Triton x-100_ is the absorbance value of the supernatant treated with Triton x-100 and A_PBS_ is the absorbance value of the supernatant treated with PBS.

**Open field test**

The open field test is employed to quantify depressive behavior in mice following burn injury. Each mouse is positioned in the corner of a box measuring 50 cm in diameter and 40 cm in height. A camera system was utilized to record the mice's movements for up to 6 minutes in a dimly lit environment. The following parameters were assessed: (1) total distance travelled; (2) average rate of movement; and (3) total distance travelled in the central area.

**Tail suspension test**

The tail suspension test was used to evaluate depression-like behaviors. In brief, testing was carried out in a darkened room with minimal background noise.Each mouse was individually suspended upside down by tail with the head 5 cm above the bottom. Mobility time was determined for the last 4 minutes during a 6 minutes test.

**Forced swimming test**

The forced swim test was also employed to evaluate depressive-like behavior in mice following burn injuries. The mice were placed in a 10 cm diameter cylindrical apparatus. The temperature of the water in the apparatus was maintained at 25°C, with a depth of 30 cm. Mice were allowed to swim for 6 minutes and mobility time was recorded during the last 4 minutes. A floating posture in which the head of the mouse remains above the water surface and shows signs of struggle is mobility.

**Histology and immunohistochemistry**

The mice were sacrificed, and the regenerated skin samples were excised and collected on day 10. The sample was fixed with paraformaldehyde (4 %) for 2 days, and then the tissues were dehydrated with sucrose (30 %) for 2-3 days and then immersed in the paraffin. 5 μm sections were prepared for Hematoxylin-eosin (H&E) and Masson trichrome (MT) staining.

For immunohistochemical (IHC) evaluation, skin wound, and brain tissue was collected on day 10 of treatment. blocked with normal goat serum, then incubated with CD31(Beyotime, 1:200, #AF1642), VEGF (Beyotime, 1:200, #AF1309), MMP-9 (Beyotime, 1:200, #AF5234), CD90 (Beyotime, 1:200, #AF1636) and BDNF (Beyotime, 1:200, #AF5234) After washed thrice with TBST, the slices were incubated with FICT or FRITC conjugated goat secondary antibody for 1 h, respectively. Finally, the slices were washed thrice with TBST, stained with DAB before sealing the surface, and observed on the upright fluorescence microscope.

**Nissl staining**

To assess the activity of neurons in the hippocampal region of the brain after burn injury, Nissl bodies in the cytoplasm of neuronal cells were stained using the Nissl kit (Beyotime, #C0117) for 10 minutes at room temperature, washed twice with ultrapure water for 5 minutes each time, and visualized under a microscope after dehydration.

**Immunofluorescence Staining**

Immunofluorescence immunoassay was used to detect antibody expression on day 10. Triton X-100 (0.5%) was used after antigen repair and closed with goat serum (5%). Subsequently, samples were incubated with anti-NF200 (Cell signaling technology, #2836S), anti-F4/80 (Cell signaling technology, 1:500, #30325T), anti-CD86 (Cell signaling technology, 1:500, #91882T), anti-MAP-2 (Abcam, 1:500, #ab32454), anti-IBA-1 (Wako, 1:500, #019-19741), and GFAP (Cell signaling technology, 1:500, #3670) at 4 °C overnight. After washed thrice with TBST, the slices were incubated with secondary antibody for 1 h, respectively. Finally, the slices were washed thrice with TBST, stained with DAPI before sealing the surface, and observed on the upright fluorescence microscope.

**Statistical analysis**

Results were analyzed by using GraphPad Prism 7 software. Differences between the two groups were assessed using unpaired *t*-tests. For multiple comparisons, statistical significance was analyzed using one-way analysis of variance (ANOVA), followed by Sidak’s post-hoc test, which was used when comparing all the conditions. The level of statistical significance was set at *p* < 0.05. ******p* < 0.05, **^#^***p* < 0.05 were considered significant, and *******p* < 0.01, ********p* < 0.001, *********p* < 0.0001, **^##^***p* < 0.01, **^###^***p* < 0.001, **^####^***p* < 0.0001 were considered highly significant. Unless otherwise indicated, all data were expressed as mean ± standard error of the mean (S.E.M).

**Supplementary Figures**


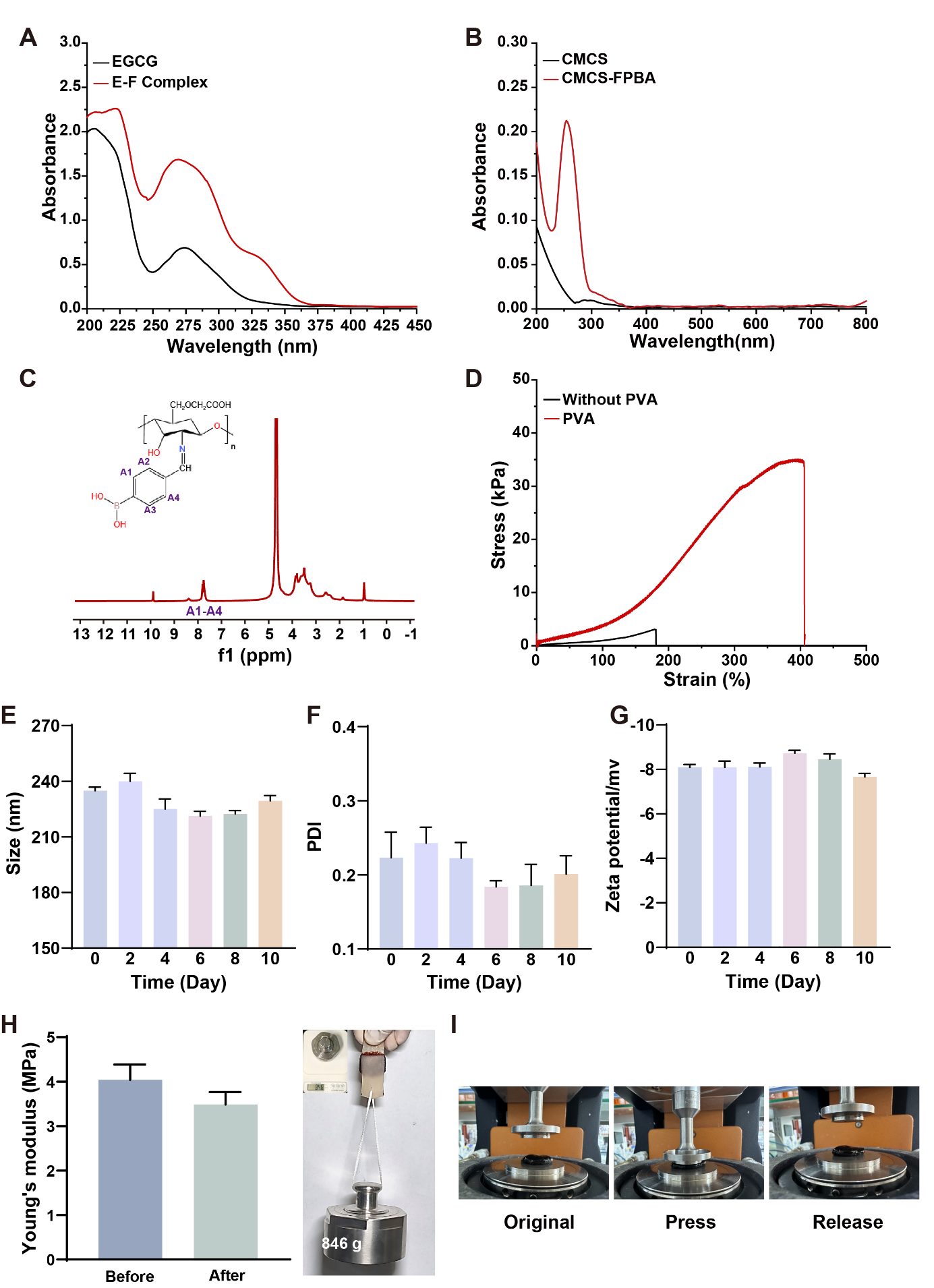


**Figure S1.** **(A)** UV-vis spectrum of the EGCG and E-F Complex solution. **(B)** UV-vis spectrum of the CMCS and CMCS-FPBA solution. **(C)** ^1^H NMR spectrum of CMCS-FPBA. **(D)** The tensile stress-strain curves of the hydrogel samples, prepared with a ratio of W_CMCS_ : W_E-F_ = 8:1, after addition of PVA. **(E)** Hydrodynamic diameters, **(F)** Polymer dispersity index and **(G)** Zeta potential of HMPB dispersed in water. Data are expressed as mean ± S.E.M. (n=3). (**H**) The changes in Young's modulus of H@EFCP over a period of 15 minutes of water absorption. Data are expressed as mean ± S.E.M. (n=3). (**I**) The original compression and recovery shapes of H@EFCP.


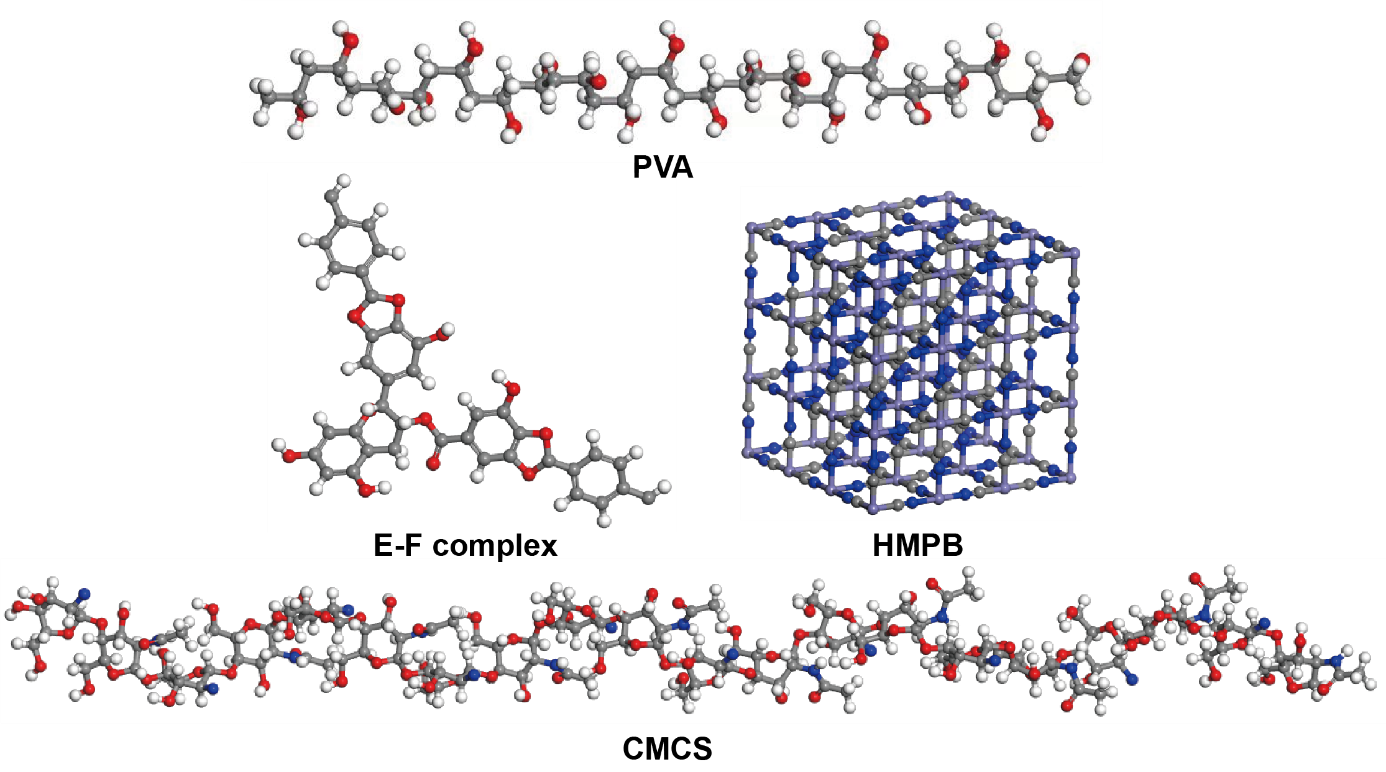


**Figure S2**. **Molecular structure of major components in molecular simulation calculations.**


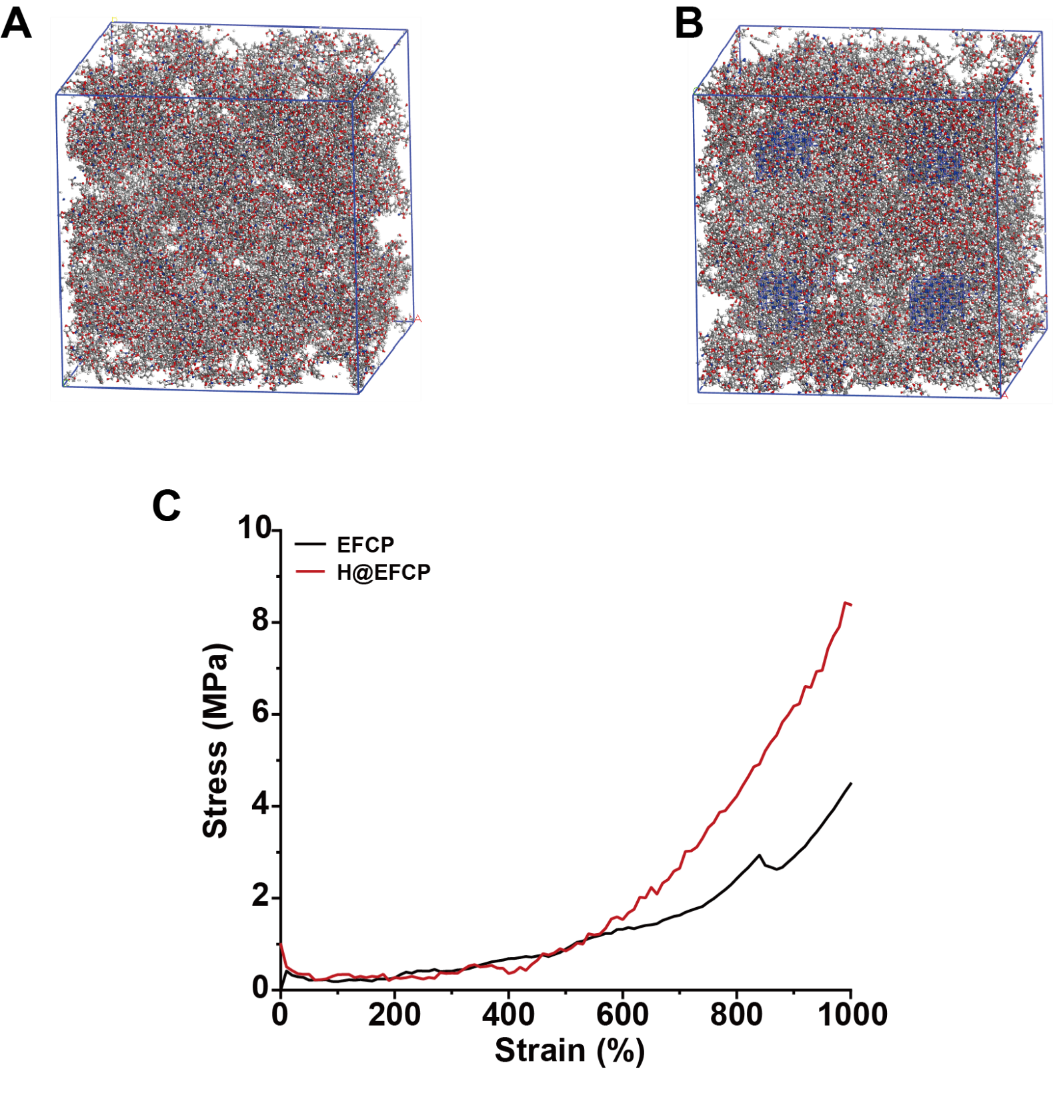


**Figure S3**. **(A)** Initial simulation configuration of EFCP. **(B)** Initial simulation configuration of H@EFCP. **(C)** The tensile stress-strain curves resulting from molecular dynamics simulations.


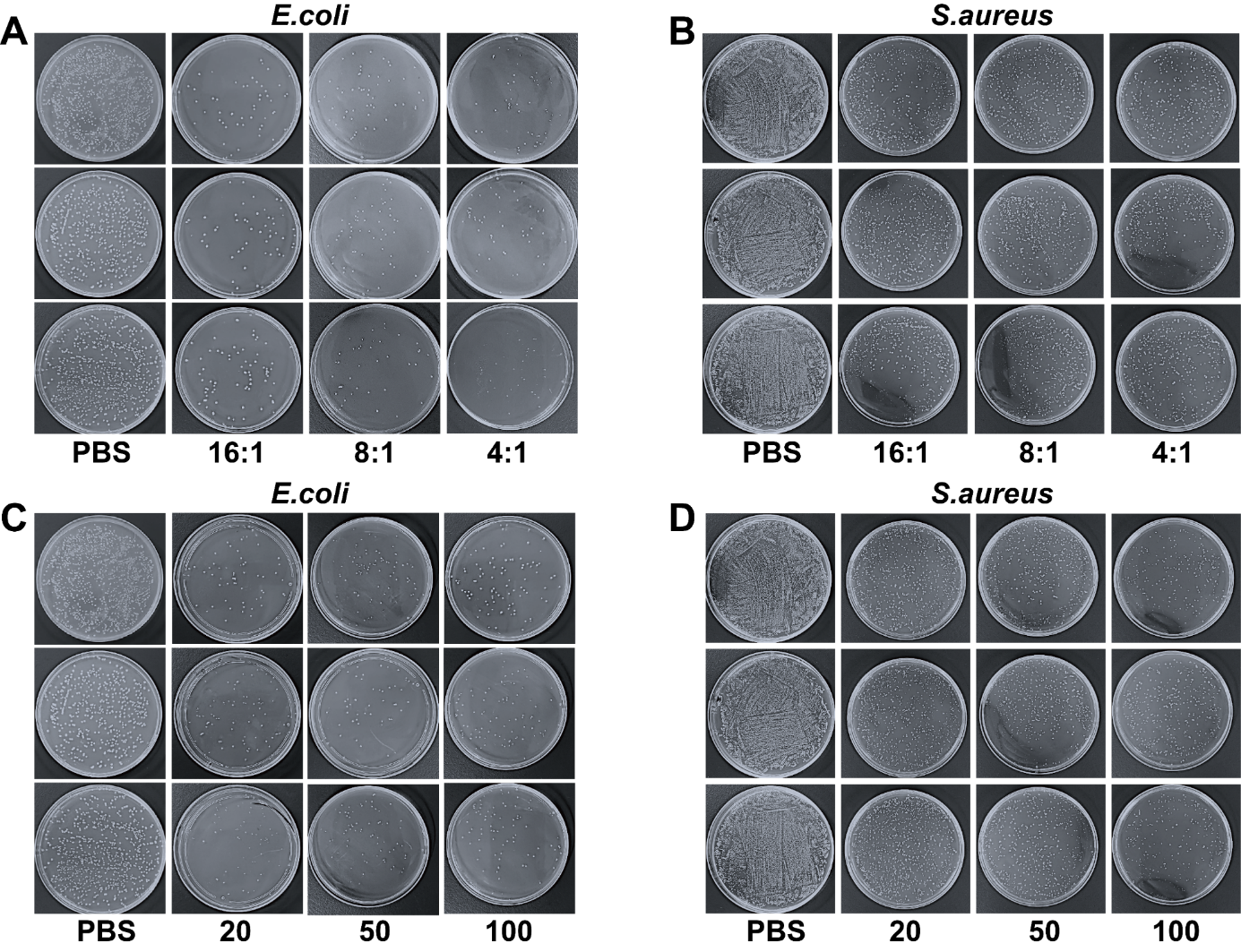


**Figure S4**. (**A-B**) Images of surviving bacterial clones (**A**) *E.coli* and (**B**) *S.aureus* on culture plates after contact with PBS and H@EFCP with different E-F complexes ratios without HMPB (W_CMCS_:W_E-F complex_ = 16:1, 8:1, and 4:1). (**C-D**) Images of bacteria (**C**) *E.coli* and (**D**) *S.aureus* exposed to PBS and H@EFCP containing different concentrations of HMPB surviving on culture plates (0 μg/mL, 20 μg/mL, 50 μg/mL and 100 μg/mL).


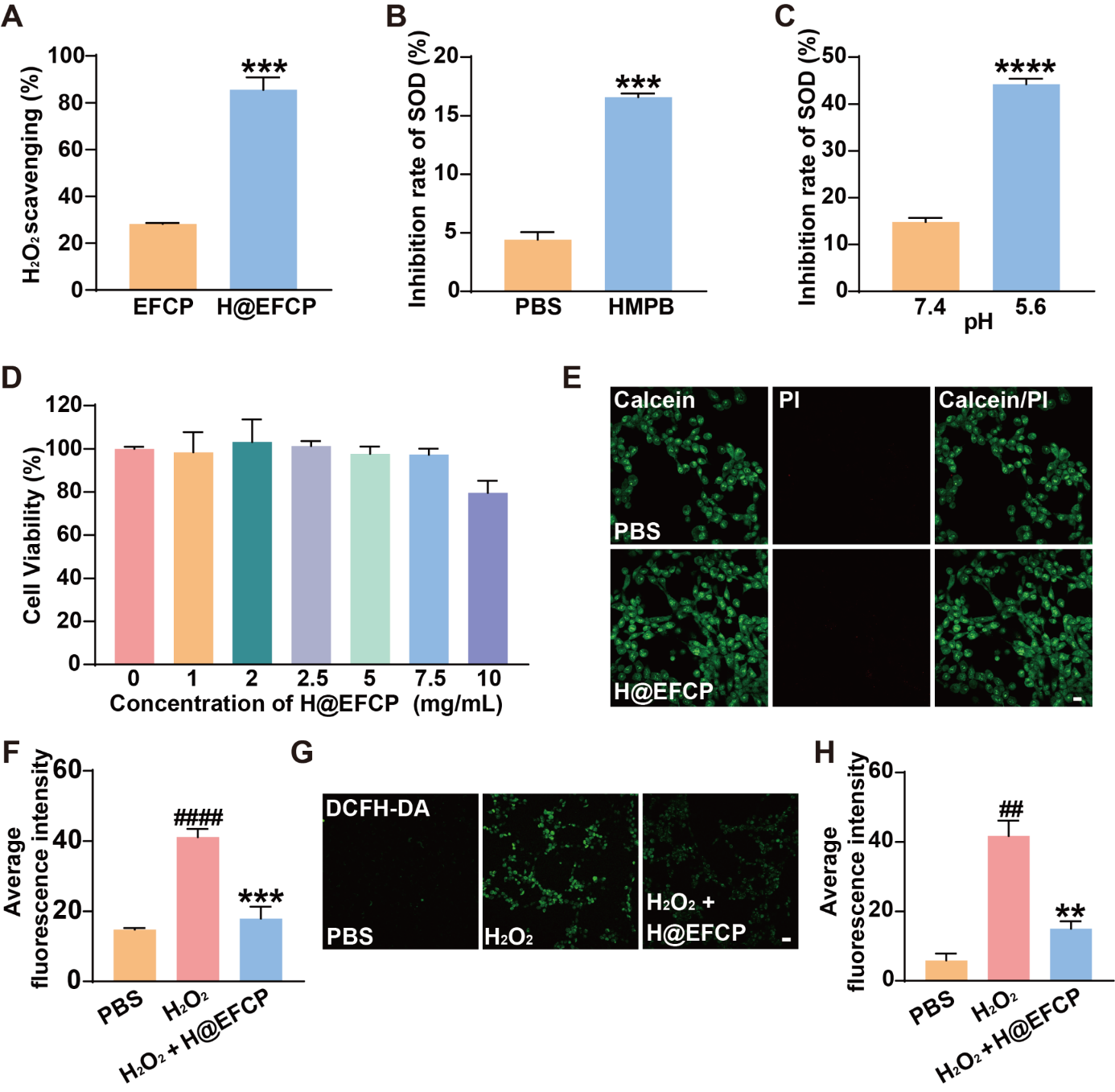


**Figure S5**. (**A**) The scavenging of H_2_O_2_ by H@EFCP and EFCP. Data are expressed as mean ± S.E.M. (n=3, two-tailed unpaired Student's *t*-test, ********p* < 0.001 vs EFCP group). (**B**) SOD-like activity of HMPB. Data are expressed as mean ± S.E.M (n=3, two-tailed unpaired Student's *t*-test, ********p* < 0.001 vs the PBS group). (**C**) SOD-like activity of H@EFCP at different pH. Data are expressed as mean ± S.E.M (n=3, two-tailed unpaired Student's *t*-test, *********p* < 0.0001 vs the pH=7.4 group). (**D**) Cell viability of NIH3T3 cells after incubation in the leaching solution with different concentrations for 48 h. Data are expressed as mean ± S.E.M (n=3). (**E**) Live/Dead staining of NIH3T3 cells after incubation in a leaching solution of H@EFCP (5 mg/mL) for 24 h. Scale bar: 20 μm. (**F**) Average fluorescence intensity of DCFH-DA. Data are expressed as mean ± S.E.M. (n=3, one-way ANOVA and Sidak's multiple comparison tests, ********p* < 0.001 vs the H_2_O_2_-treated group, **^####^***p* < 0.0001 vs the PBS group). (**G**) The alleviation of oxidative stress in SH-SY5Y was monitored via a DCFH-DA after different treatments. Scale bar, 20 μm. (**H**) Mean fluorescence intensity of DCFH-DA in SH-SY5Y cells. Data are expressed as mean ± S.E.M. (n=3, one-way ANOVA and Sidak's multiple comparison tests, *******p* < 0.01 vs the H_2_O_2_-treated group, **^##^***p* < 0.01 vs the PBS group).


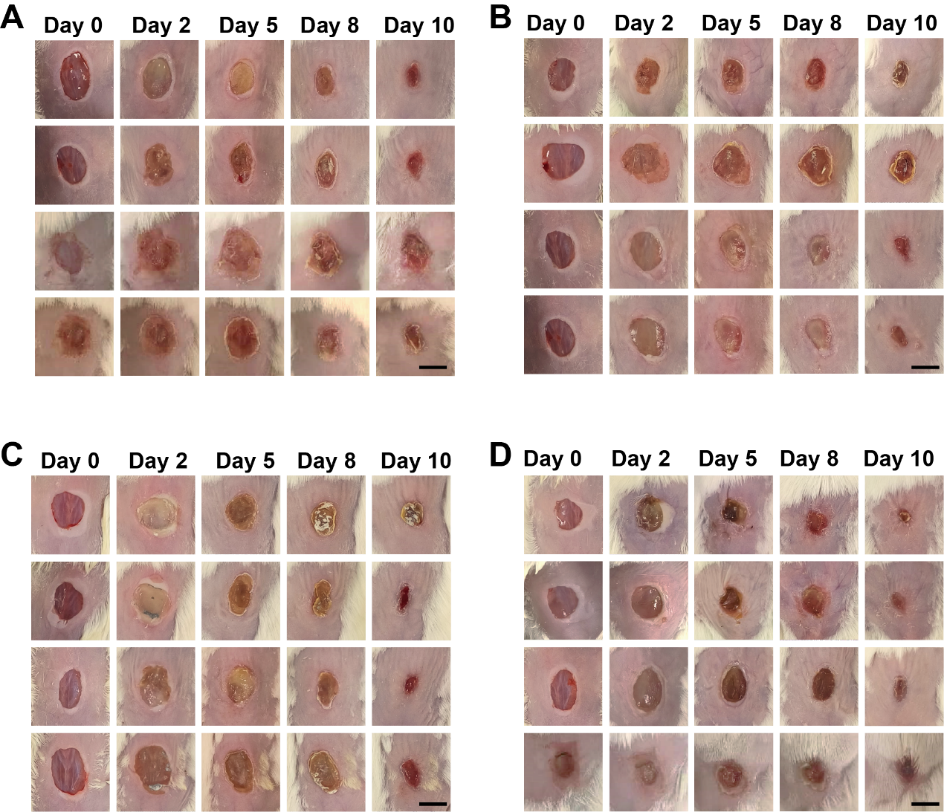


**Figure S6.** Wound images with different treatment regimens at specific time intervals. (**A**) Burn model, (**B**) FCP, (**C**) SSD and (**D**) H@EFCP. Scale bars, 3 mm.


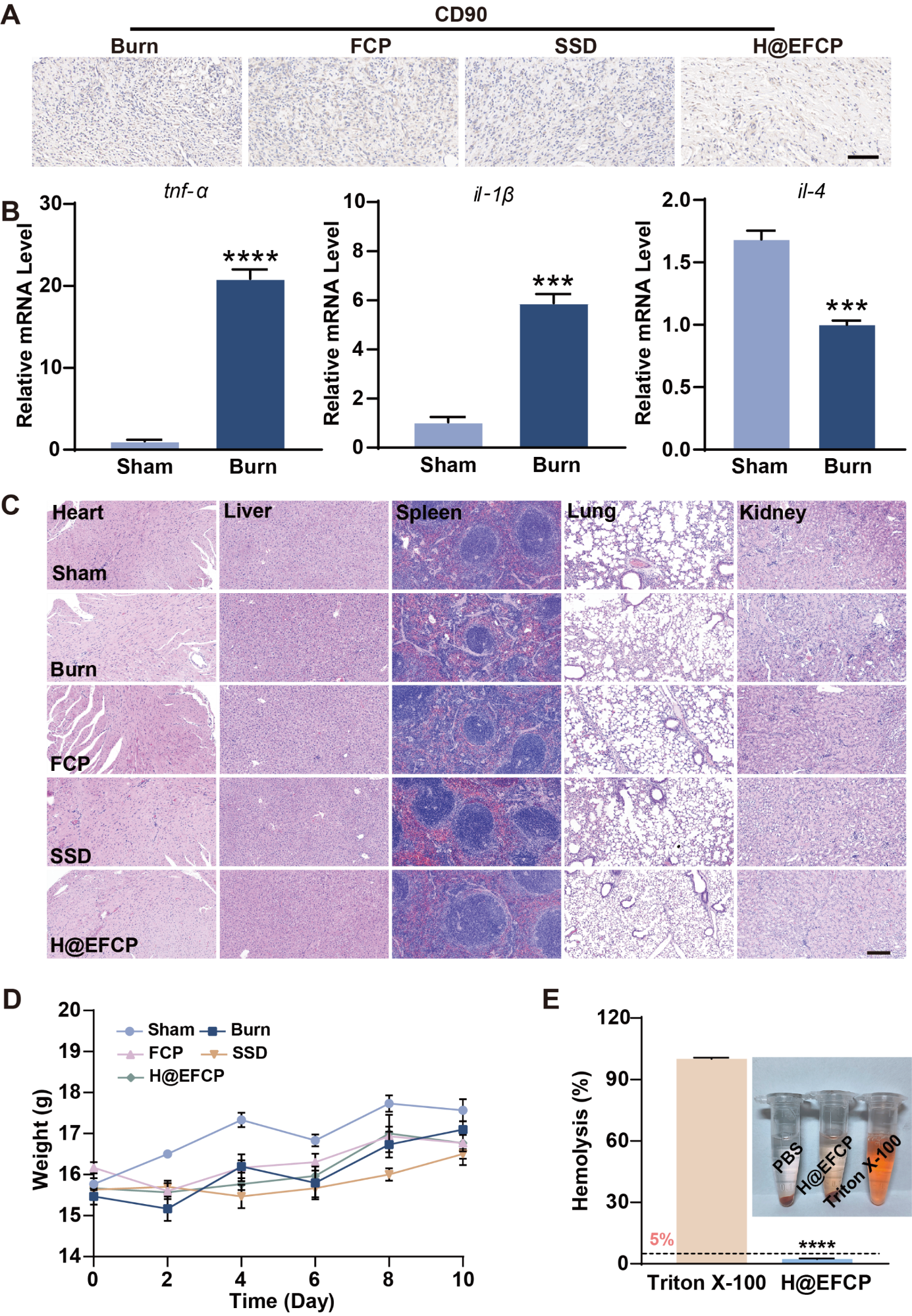


**Figure S7.** (**A**) IHC staining of CD90. Scale bar, 100 µm. (**B**) The levels of inflammatory factors in the normal and model groups. Data are expressed as mean ± S.E.M. (n=4, two-tailed unpaired Student's *t*-test, ********p* < 0.001, *********p* < 0.0001 vs the sham group). (**C**) H&E staining of heart, liver, spleen, lung and kidney in all groups on day 10. Scale bar, 100 µm. (**D**) The changes in body weight of mice in the different treatment groups. Data are expressed as mean ± S.E.M. (n=5). (**E**) The percentage of the hemolysis rate of H@EFCP. Data are expressed as mean ± S.E.M. (n=3, two-tailed unpaired Student's *t*-test, *********p* < 0.0t001 vs the Triton X-100 group).


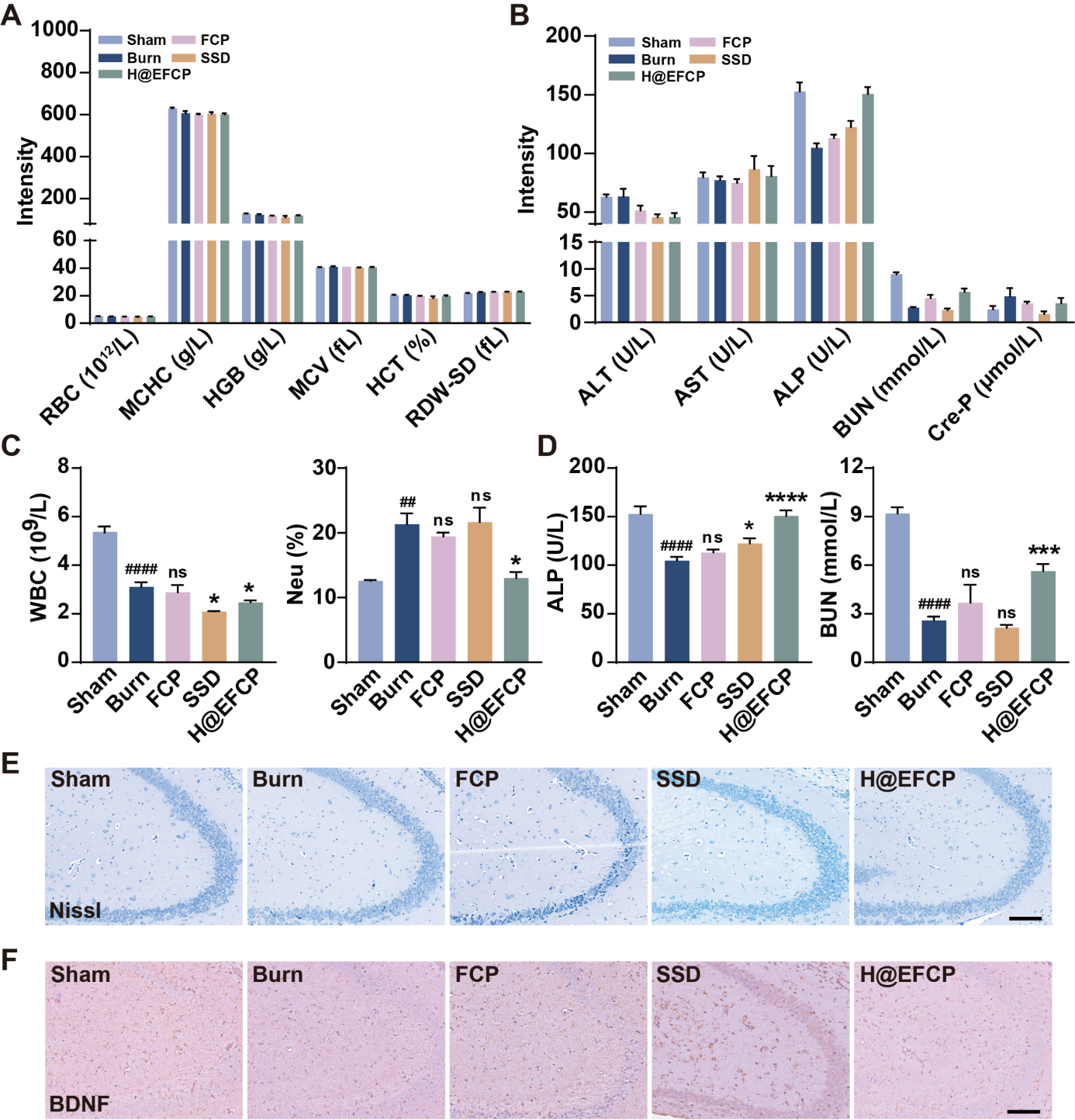


**Figure S8.** (**A**) The assessment of standard hematology markers including red blood cells (RBC), mean corpuscular hemoglobin concentration (MCHC), hemoglobin (HGB), mean corpuscular volume (MCV), hematocrit (HCT) and red blood cell volume distribution width (RDW-SD). Data are expressed as mean ± S.E.M. (n=5). (**B**) Blood biochemical analyses of ALT, alanine transaminase; AST, aspartate transaminase; ALP, alkaline phosphatase; BUN, blood urea; and Cre-P, creatinine. Data are expressed as mean ± S.E.M. (n=5). (**C**) The assessment of white blood cells (WBC) and Neutrophil (NEU). Data are expressed as mean ± S.E.M. (n=5, one-way ANOVA and Sidak's multiple comparison tests, ns, no significance, ******p* < 0.05 vs the burn group, **^##^***p* < 0.01, **^####^***p* < 0.0001 vs the sham group). (**D**) The assessment of alkaline phosphatase (ALP) and blood urea (BUN). Data are expressed as mean ± S.E.M. (n=5, one-way ANOVA and Sidak's multiple comparison tests, ns, no significance, ******p* < 0.05, ********p* < 0.001, *********p* < 0.0001 vs the burn group, **^####^***p* < 0.0001 vs the sham group.) (**E**) Nissl staining of live neurons in the CA3 regions of the hippocampus of different groups. Scale bar, 100 µm. (**F**) Analysis of hippocampal BDNF expression using immunohistochemistry slides. Scale bar, 100 µm.

**Table S1**. RT-qPCR primer sequences.

| **gene** | **Forward (5′-3)** | **Reverse (3′-5)** |
| --- | --- | --- |
| *gadph* | GGTGAAGGTCGGTGTGAACG | CTCGCTCCTGGAAGATGGTG |
| *tnf-α* | CAAAATTCGAGTGACAAGCCT | CTGGGAGTAGACAAGGTACAAC |
| *il-1β* | TGTGTAATGAAAGACGGCA | TCCACTTTGCTCTTGACGGCAC |
| *il-4* | GAGTGAGCTCGTCTGTAGGG | GAAGTCTTTCAGTGATGTGG |
| *il-6* | AGCCAGAGTCCTTCAGAGAG | CTTAGCCACTCCTTCTGTGAC |
| *sp* | TTTCTCGTTTCCACTCAACTGTT | GTCTTCGGGCGATTCTCTGC |
| *c-fos* | TACTACCATTCCCCAGCCCGA | TCCACGTTGCTGATGCTCTT |
